# A systematic study of molecular diagnosis, treatment, and prognosis in infant-type hemispheric glioma: An individual patient data meta-analysis of 164 patients

**DOI:** 10.1093/neuonc/noaf264

**Published:** 2025-11-08

**Authors:** Lara Chavaz, Aditi Bagchi, Sandeep K Dhanda, Fabienne Toutain, Stefan M Pfister, Dominik Sturm, Torsten Pietsch, Gerrit H Gielen, Andreas Waha, Matthew Clarke, Congyu Lu, Michael Karremann, Martin Benesch, Thomas Perwein, Gunther Nussbaumer, Christof Kramm, Maura Massimino, Veronica Biassoni, Maria Vinci, Angela Mastronuzzi, Dannis van Vuurden, Sophie E M Veldhuijzen van Zanten, Alan Mackay, Chris Jones, David T W Jones, Ana S Guerreiro Stucklin, Uri Tabori, Cynthia Hawkins, Scott Ryall, Andrés Morales La Madrid, Alvaro Lassaletta, Simon Bailey, Darren Hargrave, Jason Chiang, Moatasem El-Ayadi, Bruna Minniti Mançano, Rui Manuel Reis, Christian Hagel, Hamza Gorsi, Nicolas Silvestrini, Ahmed Gilani, Ludmila Papusha, Paul Klimo Jr, Xin Zhou, Amar Gajjar, Giles W Robinson, Andre O von Bueren

**Affiliations:** Department of Pediatrics, Gynecology and Obstetrics, Division of Pediatric Hematology and Oncology, University Hospital of Geneva, Geneva (L.C., F.T., A.O.v.B.); Cansearch Research Platform for Pediatric Oncology and Hematology, Faculty of Medicine, Department of Pediatrics, Gynecology and Obstetrics, University of Geneva, Geneva (L.C., F.T., A.O.v.B.); Department of Oncology, Division of Neuro-Oncology, St. Jude Children’s Research Hospital, Memphis (A.B., S.K.D., A.G., G.W.R.); Department of Oncology, Division of Neuro-Oncology, St. Jude Children’s Research Hospital, Memphis (A.B., S.K.D., A.G., G.W.R.); Department of Pediatrics, Gynecology and Obstetrics, Division of Pediatric Hematology and Oncology, University Hospital of Geneva, Geneva (L.C., F.T., A.O.v.B.); Cansearch Research Platform for Pediatric Oncology and Hematology, Faculty of Medicine, Department of Pediatrics, Gynecology and Obstetrics, University of Geneva, Geneva (L.C., F.T., A.O.v.B.); Department of Pediatric Oncology, Hematology & Immunology, Heidelberg University Hospital, Heidelberg (S.M.P., D.S.); Division of Pediatric Neuro-Oncology, Hopp Children’s Cancer Center Heidelberg (KiTZ), Heidelberg (S.M.P.); German Cancer Research Center (DKFZ) and National Center for Tumor Diseases (NCT), NCT Heidelberg, A Partnership Between DKFZ and Heidelberg University Hospital, Heidelberg (S.M.P., D.S., D.T.W.J.); Department of Pediatric Oncology, Hematology & Immunology, Heidelberg University Hospital, Heidelberg (S.M.P., D.S.); Division of Pediatric Glioma Research, Hopp Children’s Cancer Center Heidelberg (KiTZ), Heidelberg (D.S., D.T.W.J.); German Cancer Research Center (DKFZ) and National Center for Tumor Diseases (NCT), NCT Heidelberg, A Partnership Between DKFZ and Heidelberg University Hospital, Heidelberg (S.M.P., D.S., D.T.W.J.); Institute of Neuropathology, DGNN Brain Tumor Reference Center, University of Bonn, Bonn (T.P., G.H.G., A.W.); Institute of Neuropathology, DGNN Brain Tumor Reference Center, University of Bonn, Bonn (T.P., G.H.G., A.W.); Institute of Neuropathology, DGNN Brain Tumor Reference Center, University of Bonn, Bonn (T.P., G.H.G., A.W.); Division of Molecular Pathology, Institute of Cancer Research, London (M.C., A.M., C.J.); Department of Computational Biology, St. Jude Children’s Research Hospital, Memphis (C.L., X.Z.); Department of Pediatric and Adolescent Medicine, University Medical Center Mannheim, Medical Faculty Mannheim, Heidelberg University, Mannheim (M.K.); Division of Pediatric Hematology and Oncology, Department of Pediatrics and Adolescent Medicine, Medical University of Graz, Graz (M.B., T.P., G.N.); Division of Pediatric Hematology and Oncology, Department of Pediatrics and Adolescent Medicine, Medical University of Graz, Graz (M.B., T.P., G.N.); Division of Pediatric Hematology and Oncology, Department of Pediatrics and Adolescent Medicine, Medical University of Graz, Graz (M.B., T.P., G.N.); Division of Pediatric Hematology and Oncology, University Medical Center Göttingen, Göttingen (C.K.); Pediatric Oncology Unit, Fondazione IRCCS Istituto Nazionale dei Tumori di Milano, Milan (M.M., V.B.); Pediatric Oncology Unit, Fondazione IRCCS Istituto Nazionale dei Tumori di Milano, Milan (M.M., V.B.); Research Area of Onco-Hematology and Pharmaceutical GMP Facility, Bambino Gesù Children’s Hospital IRCCS, Rome (M.V., A.M.); Research Area of Onco-Hematology and Pharmaceutical GMP Facility, Bambino Gesù Children’s Hospital IRCCS, Rome (M.V., A.M.); Princess Máxima Center for Pediatric Oncology, Utrecht (D.v.V.); Department of Radiology and Nuclear Medicine, Erasmus MC, Rotterdam (S.E.M.V.v.Z.); Division of Molecular Pathology, Institute of Cancer Research, London (M.C., A.M., C.J.); Division of Molecular Pathology, Institute of Cancer Research, London (M.C., A.M., C.J.); Division of Pediatric Glioma Research, Hopp Children’s Cancer Center Heidelberg (KiTZ), Heidelberg (D.S., D.T.W.J.); German Cancer Research Center (DKFZ) and National Center for Tumor Diseases (NCT), NCT Heidelberg, A Partnership Between DKFZ and Heidelberg University Hospital, Heidelberg (S.M.P., D.S., D.T.W.J.); Department of Oncology and Children’s Research Center, University Children’s Hospital Zurich, Zurich (A.S.G.S.); The Arthur and Sonia Labatt Brain Tumour Research Centre, The Hospital for Sick Children, Toronto (U.T., C.H., S.R.); Department of Hematology Oncology, The Hospital for Sick Children, Toronto (U.T.); The Arthur and Sonia Labatt Brain Tumour Research Centre, The Hospital for Sick Children, Toronto (U.T., C.H., S.R.); Division of Pathology, Hospital for Sick Children, Toronto (C.H.); The Arthur and Sonia Labatt Brain Tumour Research Centre, The Hospital for Sick Children, Toronto (U.T., C.H., S.R.); Pediatric Cancer Center Barcelona, Hospital Sant Joan de Deu, Barcelona (A.M.L.M.); Department of Pediatric Hematology Oncology, Hospital Infantil Universitario Niño Jesús, Madrid (A.L.); Department of Paediatric Oncology, Sir James Spence Institute of Child Health, Royal Victoria Infirmary Queen, Newcastle upon Tyne (S.B.); Pediatric Oncology Unit, Great Ormond Street Hospital for Children, London (D.H.); Department of Pathology, St. Jude Children’s Research Hospital, Memphis (J.C.); Department of Pediatric Oncology, National Cancer Institute, Cairo University, Cairo (M.E.-A.); Departament of Pediatric Oncology, Barretos Cancer Hospital, Barretos (B.M.M.); Molecular Oncology Research center, Barretos Cancer Hospital, Barretos (R.M.R.); Life and Health Research Institute (ICVS), Medical School, University of Minho, Braga (R.M.R.); Institute of Neuropathology, University Medical Center Hamburg-Eppendorf, Hamburg (C.H.); Department of Hematology/Oncology, Children’s Hospital of Michigan, Detroit (H.G.); Central Michigan University School of Medicine, Mt Pleasant (H.G.); Clinical Research Platform—Paediatrics, Gynaecology and Obstetrics, Department of Women, Child, and Adolescent Medicine, Geneva University Hospitals and Faculty of Medicine, Geneva (N.S.); Department of Pathology, Children’s Hospital Colorado, Aurora (A.G.); Dmitry Rogachev National Medical Research Center of Pediatric Hematology, Oncology and Immunology, Moscow (L.P.); Department of Surgery, St. Jude Children’s Research Hospital, Memphis (P.K.); Department of Neurosurgery, University of Tennessee Health and Science Center, Memphis (P.K.); Le Bonheur Neuroscience Institute, LeBonheur Children’s Hospital, Memphis (P.K.); Department of Computational Biology, St. Jude Children’s Research Hospital, Memphis (C.L., X.Z.); Department of Oncology, Division of Neuro-Oncology, St. Jude Children’s Research Hospital, Memphis (A.B., S.K.D., A.G., G.W.R.); Department of Oncology, Division of Neuro-Oncology, St. Jude Children’s Research Hospital, Memphis (A.B., S.K.D., A.G., G.W.R.); Department of Pediatrics, Gynecology and Obstetrics, Division of Pediatric Hematology and Oncology, University Hospital of Geneva, Geneva (L.C., F.T., A.O.v.B.); Cansearch Research Platform for Pediatric Oncology and Hematology, Faculty of Medicine, Department of Pediatrics, Gynecology and Obstetrics, University of Geneva, Geneva (L.C., F.T., A.O.v.B.)

**Keywords:** high-grade glioma (HGG), infant-type hemispheric glioma (IHG), pediatric-type diffuse high-grade glioma (pHGG), receptor tyrosine kinase (RTK), tyrosine kinase inhibitor (TKI)

## Abstract

**Background:**

Due to the novelty and rarity of infant-type hemispheric glioma (IHG), optimal treatment and factors determining clinical outcomes are yet to be established.

**Methods:**

We curated a series of 164 patients with IHG; 155 identified by methodical literature search and nine additional patients contributed by collaborators.

**Results:**

All tumors were hemispheric, diagnosed at a median age of 3.4 (0-52) months, and frequently (95%) non-metastatic. One hundred forty-two (86.5%) tumors harbored fusions involving receptor tyrosine kinase (RTK) genes (*ALK* [67/142, 47%], *NTRK*1/2/3 [32/142, 22.5%], *ROS1* [29/142, 20.4%], *MET* [13/142, 9.2%], and *ABL2* [1/142, 0.7%]). Sixty-four percentage, 20%, and 8% of patients were treated with surgery and adjuvant chemotherapy, surgery-only, and surgery plus targeted therapy, respectively. Five patients received radiation. Three-year event-free survival (EFS) and overall survival (OS) was 49.5% [40.7-60.2] and 79.6% [72.1-87.9], respectively. Twenty-two patients succumbed to disease, of which tumor progression (8/22, 36%) and intra-cranial hemorrhage (5/22, 23%) were the most common causes. Multivariate analysis showed that the factors most associated with an increased risk of death were no treatment except for surgery and presence of residual tumor after definitive surgery. These findings present a challenging dichotomy where surgery is both a serious risk factor for early death and, when successful, a benefit.

**Conclusions:**

Together, these findings show that IHG is a fusion driven tumor of the very young that is survivable even after progression. While optimal primary therapy for patients with IHG has yet to be established, the findings of this meta-analysis suggest treatment should focus on lowering surgical morbidity and improving its success.

Key Points

- Infant-type hemispheric gliomas frequently harbor oncogenic RTK fusions, most commonly ALK.
- While complete resection improves survival, upfront neurosurgery increases intracranial bleeding and early mortality.
- Development of safer surgical protocol may be key to optimize outcomes.

Importance of the Study Infant-type hemispheric glioma are rare brain tumor of infancy and are oncogenically driven by RTK fusion. Despite a high overall survival rate with radiation sparing treatment, the course is fraught with morbid primary surgeries, early deaths from hemorrhagic complications, and progression of tumor during or immediately after primary treatment, often requiring multiple lines of therapy for the cure. This meta-analysis is the first systematic effort to study a large cohort of IHG patients to develop an understanding of the factors that affect the clinical outcome. We report two factors most associated with an increased risk of primary treatment failure; (1) no treatment except for surgery and (2) presence of residual tumor after definitive surgery. These results underscore a clinical challenge—where the complete resection that is critical for cure simultaneously poses a high risk of early death, indicating that treatment should focus on reducing the morbidity of definitive surgery of patients.

Infant-type hemispheric glioma (IHG) is a newly defined tumor of the central nervous system (CNS) within pediatric-type diffuse high grade glioma (pHGG) in the WHO 2021 classification of the CNS tumors.^1^^,^^2^ While other classes of pHGG (diffuse midline glioma, H3K27-altered, diffuse hemispheric glioma, H3G34-mutant, and pHGG H3 wild type and *IDH* wild type) are oncogenically driven by genomic alterations in genes like *H3C1, H3C3, MYCN, PDGFRA, EGFR,* etc.,^3–6^ IHG harbor unique chromosomal rearrangements involving receptor tyrosine kinase (RTK)-coding genes like neutrophic *tyrosine receptor kinase 1/2/3 (NTRK1/2/3), ROS proto-oncogene 1 receptor tyrosine kinase (ROS1), anaplastic lymphoma kinase (ALK),* and *MET proto-oncogene receptor tyrosine kinase (MET).*^7–9^ These alterations often lead to different 5’ binding partners fusing with 3’ end of the truncated RTK with the kinase domain leading to the formation of chimeric proteins that constitutively activate the downstream PI3K/AKT and MAPK/ERK pathways critical for cellular growth and proliferation.^7–9^

Historically high-grade gliomas (HGG) were considered biologically and clinically homogenous due to their histo-morphological similarities, therefore, uniform treatment with primary surgery followed by involved field radiation therapy (RT) was considered as standard. As RT is detrimental to early growth and development, HGG diagnosed in younger children (0-3 years) were treated with surgery and adjuvant chemotherapy to delay, avoid, or dose reduce RT. Despite the treatment modification, the infants, unexpectedly, had better survival rates and several patients were cured without RT (Overall survival [OS] 50%-70%).^9–15^

This contrasting clinical outcome suggested that HGGs diagnosed at very young age were intrinsically different. This was confirmed by subsequent molecular studies reporting different genomic drivers in infantile HGGs and their epigenetic segregation from HGGs diagnosed in older age groups.^7^^,^^9^ These discoveries were instrumental in recategorization of gliomas in the 5th edition of classification of CNS tumors integrating molecular characteristics of HGGs with histo-morphological features and evolution of IHG as a unique diagnostic tumor type within pHGG.^1^^,^^2^ Furthermore, molecular discoveries of RTK fusions (*NTRK1/2/3*, *ROS1*, *ALK*, *MET*) as oncogenic drivers provided the rationale for using tyrosine kinase inhibitors (TKIs) (Entrectinib, Larotrectinib, Lorlatinib, Alectinib, etc.) in treatment.^7^ ^,^^8^^,^^16–18^

The progress in standardizing clinical management of patients with IHG lags behind the remarkable gains made in our biological understanding of the tumor. The ultra rarity of diagnosis, the lack of frontline clinical trials, and the absence of patient registries specific to IHG have prevented systematic curation, review, and study of IHG in a large enough sample size to identify representative characteristics affecting prognosis and treatment. Currently, treatment often follows the historical convention of maximal surgical resection with adjuvant chemotherapy despite evolving preclinical and clinical data that suggest patients with IHG are good candidates for molecular directed therapy.^7^^,^^16^ Several clinical studies have published safety and efficacy data on molecular directed treatments in pediatric patients with fusion positive CNS tumors including IHGs.^18–20^ Still, molecular directed therapy is often reserved for recurrent setting or offered as a palliative option to patients.

This metadata analysis is the first systematic effort to curate a large series of unpublished and published patients with IHG validated by established diagnostic criteria specified in the 5th edition of the WHO classification of CNS tumors. The primary objective of the meta-analysis is to identify clinically significant factors that may inform prognosis and survival outcomes in patients and to further advance our understanding of the molecular landscape, clinical course, and natural history of this very rare brain tumor diagnosed during infancy.

## Methods

Comprehensive literature search was conducted using medical subject headings (MeSH) within PubMed and EMBASE without date restriction on June 15, 2024. For PubMed the MeSH search terms were “infant,” “hemispheric,” “glioma,” “high-grade glioma,” “case”; 180 (“Infant” AND “high-grade glioma”), 127 ((Infan*) AND (hemispheric) AND (glioma*)) and 40 (“Infant” AND “high-grade glioma” AND “case”). English language and studies with human subjects were set as limitations. For EMBASE the search term “infant-type hemispheric glioma” was used, and 69 results were obtained (**Table S1**).

**Figure 1.**
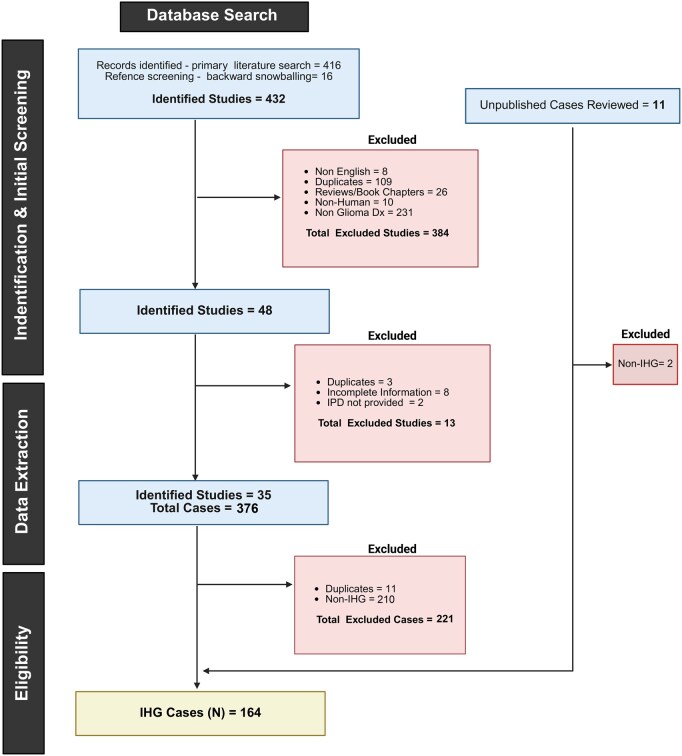
The Preferred Reporting Items for Systematic Reviews and Meta-Analyses (PRISMA) flow diagram summarizes the identification and screening process for the articles and cases included in the study (Adapted from Page MJ, McKenzie JE, Bossuyt PM, Boutron I, Hoffman TC, Mulrow CD, et al. Al and Moher D, Shamseer L, Clarke M, Ghersi D, Liberati A, Petticrew M, Shekelle P, Stewart LA, et al.). Abbreviations: Dx: Diagnosis; IHG: Infant-Type Hemispheric Glioma; IPD: Individual Patient Data.

Of the 416 identified studies, we performed a backward snowballing search using references from 32 studies (**Table S2**), 9/32 had at least one previously unidentified study in their respective reference list, 16 additional studies were identified from the reference lists for further screening (**Table S3**) (**Figure 1**).

### Identification and Initial Screening

Two independent authors (L.C., A.O.V.B.) manually screened all 432 studies; 384 studies were excluded based on (1) Non-English language of publication (*n* = 8), (2) duplicate studies (*n* = 109), (3) studies without clinical information such as reviews and book chapters (*n* = 26), (4) studies with non-human subjects (*n* = 10), and (5) studies with non-glioma diagnosis, that were unlikely to be IHG (*n* = 231). After exclusion, 48 studies were identified for case specific data extraction (**Figure 1**) (**Table S4**).

### Data Extraction

Thirty-five studies had the desired information available in methods, results, and supplementary sections. The corresponding authors for 14 studies were contacted to obtain additional details on individual patient clinical and molecular data (IPD) (**Tables S4 and S5**).^21–23^ Individual patient data were subjected to data checking in case incoherence with the original article. L.C., F.T., A.B., and A.O.V.B. participated in data extraction process, based on a predefined template of categories including details of the study (author, date of publication, study design [case report, case series, retrospective studies, prospective trials], number of patients, clinical details of the patients [primary diagnosis, treatment, and clinical course, follow-up duration, disease status, and survival status], and available molecular characteristics of the tumor [eg, DNA and RNA sequencing, DNA methylation data, fluorescence in situ hybridization, and immunohistochemistry]). Eight additional studies were further excluded based on incomplete information. Three studies were suspected to have duplicate cases based on their case description, genetic fusion and treatment; these three studies were excluded after confirmation of duplicate cases with respective corresponding authors (**Table S4**). Among the studies (*n* = 14) for which IPD was requested, IPD was not provided for two studies (*n* = 2) (**Figure 1**).

#### Eligibility

**Figure 2.**
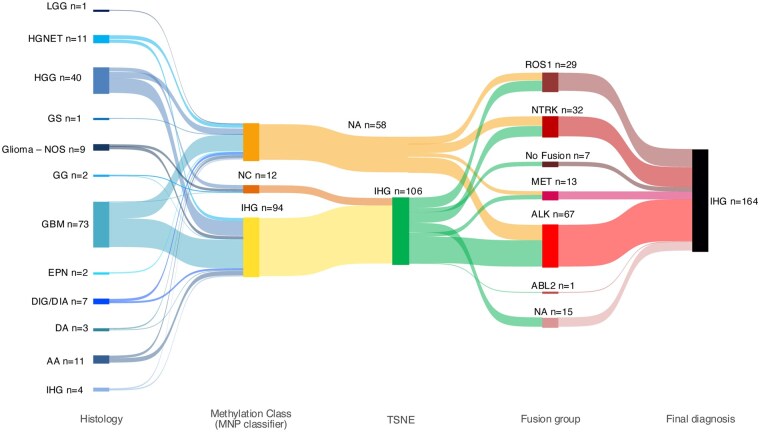
Sankey’s Plot demonstrating the integration of original histological diagnosis and molecular features used to formulate the integrated diagnosis of infant-type hemispheric glioma. Abbreviations: LGG: Low-grade glioma; HGNET: High-grade Neuroepithelial Tumor; HGG: High-grade glioma; GS: Gliosarcoma; Glioma-NOS: Glioma—Not Otherwise Specified; GG: Ganglioglioma; GBM: Glioblastoma; EPN: Ependymoma; DIG/DIA: Desmoplastic Infantile Ganglioglioma/Desmoplastic Infantile Astrocytoma; DA: Diffuse astrocytoma; AA: Anaplastic Astrocytoma; IHG: Infant-Type Hemispheric Glioma; NC: No Methylation Class; NA: Not Available; t-SNE: t-distributed stochastic neighbor embedding.

The data extraction collated 387 cases (11 unpublished cases and 376 already published cases from 35 studies) which were further screened using the 2021 WHO classification diagnostic criteria for IHG prior to inclusion in the meta-analysis. All the included tumors were newly diagnosed glial tumors located supratentorially in the cerebral hemispheres and had an original histological diagnosis compatible with IHG (**Figure 2** and **Table S7**), further, the tumors either had a distinct DNA methylation array profile indicative of IHG and/or an RTK fusion. During the molecular analysis we identified eight additional cases with same identifying labels in their FASTQ and iDAT files, these cases were also removed as duplicate cases from further analysis. One hundred and fifty-five (155) published cases and nine (9) unpublished cases met the diagnostic criteria of IHG. The final cohort used for this meta-analysis study consisted of 164 patients (**Figures 1 and 2** and **Table S7**). The unpublished cases were collected by request from co-authors who were also contacted for IPD. Two authors provided encrypted information on 11 potential cases of IHG, 2/11 cases were excluded as they did not meet the diagnostic criteria for IHG.

### Molecular Analysis

Ninety-three (93/164) iDAT files were obtained, these included tumors with either a calibrated classification score > 0.9 on the MNP classifier (*n* = 81), and 12 tumors with subthreshold scores for IHG on MNP classifier, which were included for re-analysis and confirmation of DNA methylation profiles. These were either downloaded from European Genome Phenome archive or procured by contacting the corresponding authors of respective publications. Nucleic acid (RNA/WGS) sequencing FASTQ files or the coordinates of the reported fusions were available for 84/164 tumors.

For comparable t-distributed stochastic neighbor embedding (t-SNE), methylation data points from publicly available well-characterized reference CNS tumor methylation profiles were obtained from published brain tumor datasets.^24^ Tumors were also classified using the Molecular Neuropathology (MNP) brain tumor classifier (versions 12.5 and 12.8) (www.molecularneuropathology.org) (**Table S5**). The RNA-seq data were aligned to the human reference genome (build GRCh38), as previously described.^25^^,^^26^ Fusion detection was performed using CICERO,^27^ Arriba v2.1.0,^28^ and Star Fusion^29^ with default parameters and then manually visualized and reviewed using Fusion Editor (https://proteinpaint.stjude.org/FusionEditor/).

### Statistical Analysis

Descriptive statistics were used to analyze patients’ characteristics. We defined overall survival (OS) as time from date of diagnosis to death of any cause; for patients who were alive at the time of last follow-up were censored at that date. Event-free survival (EFS) was defined as time from diagnosis to the earliest of occurrence of any of the following events: tumor progression, tumor relapse, development of second malignancy, or death from any cause. Patients who had not experienced any of these events by the time of last follow-up were censored at that date. Absence of residual disease was defined as patients who received gross total resection (GTR) or a near total resection (NTR) as part of the first line treatment. Presence of residual disease was defined as patients who received subtotal resection (STR) or biopsy (B) or no surgery as part of first line treatment.

The clinical and molecular data was used to conduct an IPD pooled analysis. EFS and OS were estimated using the Kaplan-Meier method. Differences between pairs of survival curves were assessed with the log-rank test, a non-parametric approach for comparing survival distributions between groups. Kaplan-Meier curves were generated with the survdiff() function from the R survival package.^30^ Univariate and multivariate cox-regression survival analyses were performed for the following variables: age, sex, presence of or type of RTK fusion, extent of resection, and type of primary treatment. The results were given in hazard ratio (HR), 95% CI, and *P*-value and calculated via the coxph () function in the same package (https://www.r-project.org, RRID: SCR_001905). Given the retrospective study design, no statistical endpoint was set for this analysis; therefore, all *P*-values are to be considered explorative. The local significance level was set at 0.05.

## Results

### Molecular Characterization and IHG Diagnosis

**Figure 3.**
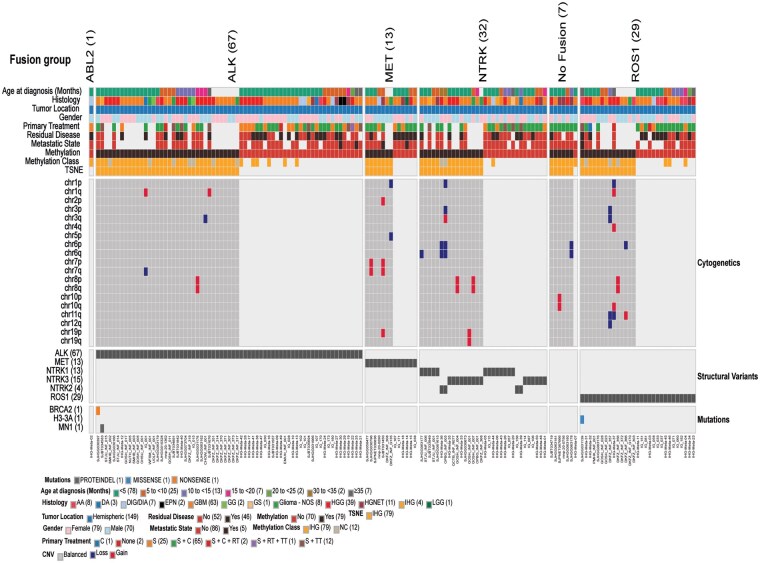
Oncoplot depicting methylation class, t-SNE class assignment, structural variants, cytogenetics, single nucleotide variants, histology, metastatic state, tumor location, age at diagnosis and gender of 164 patients with IHG.

All 164 tumors met the diagnostic criteria of IHG as defined by 2021 WHO classification of CNS Tumors (**Figure 2**).^1^^,^^2^ A total 94/164 (57.3%) tumors had a calibrated classification score > 0.9 on the MNP classifier (Version 12.5 and 12.8), 12/164 (7.3%) tumors had subthreshold scores for IHG on MNP classifier but clustered closely with IHG on t-SNE (**Figure S1**) in our re-analysis and therefore were classified as IHG. Fifty-eight tumors (58/164, 35.4%) did not have available methylation class but met the diagnostic criteria of IHG based on histology and structural rearrangements in RTKs (**Figures 2 and 3**, **Table S7**).

One hundred and forty-nine (149/164, 90.8%) cases had available data on fusion status of the tumors, *ALK* fusion was the most prevalent. One hundred and forty-two (142/149, 95.3%) tumors harbored fusions involving RTK genes (*ALK* [67/142, 47.2%], *NTRK*1/2/3 [32/142, 22.5%], *ROS1* [29/142, 20.4%], *MET* [13/142, 9.2%], and *ABL2* [1/142, 0.7%]) (**Figure 3**). Seven cases (7/149, 4.7%) with available RNA seq data were noted to be fusion negative and were diagnosed as IHG based on methylation and histology. Among the 67 *ALK* fusions *PPP1CB::ALK* (18/67, 26.9%) was the most common partner, followed by *CCDC88A::ALK* (11/67, 16.4%), *EML4::ALK* (7/67, 10.4%), *SPECC1L::ALK* (4/67, 6%), (2/67, 3%) each for *CLIP2::ALK*, *QKI::ALK*, and *SOX5::ALK* (14/67, 20.9%) had miscellaneous fusion partners and 7/67 (10.4%) were reported as *ALK* gene re-arrangements with no identified fusion partners. Among the 32 *NTRKs* fusions *ETV6::NTRK3* was the most common fusion (15/32, 46.9%), followed by *TPM3::NTRK1* (6/32, 18.8%), *TPR::NTRK1* (2/32, 6.3%) and the remaining 9/32 (28.1%) had miscellaneous fusion partners with *NTRK1/2/3*. Among the 29 *ROS1* fusions *GOPC::ROS1* was the most common fusion (9/29, 31.0%), followed by *ZCCHC8::ROS1* (4/29, 13.8%), 8/29 (27.6%) were cases reported *ROS1* genetic re-arrangements with no identified fusion partners, 3/29 (10.3%) fusion partners could not be identified, 5/29 (17.2%) had miscellaneous fusion partners. Among the 13 *MET* fusions *TRIM24::MET* (4/13, 30.8%) and *CLIP2::MET* (4/13, 30.8%) were the most common fusions, and the remaining five tumors (5/13, 38.4%) had miscellaneous fusion partners (**Table S7**).

### Clinical Characteristics

Of the 164 patients, 76/164 (46.3%) were male and 88/164 (53.7%) were female (**Table 1**, **Table S7**). One hundred and forty-three (143/164, 87.2%) patients had age of diagnoses data available, median age of diagnosis was 3.4 months (range: 0-52 months) (**Table 1**, **Table S7**). All 164 (100%) tumors were hemispheric in location and of the 93/164 (56.7%) patients for whom metastatic status was available 94.6% (88/93) were non-metastatic at diagnosis (**Table 1**, **Table S7**). The original diagnoses were glioblastoma (GBM) 73/164 (44.5%), HGG 40/164 (24.4%), anaplastic astrocytoma (AA) 11/164 (6.7%), high-grade neuroepithelial tumor (HGNET) 11/164 (6.7%), glioma not otherwise specified (Glioma-NOS) 9/164 (5.5%), desmoplastic infant glioma (DIG)/desmoplastic infant astrocytoma (DIA) 7/164 (4.3%), diffuse astrocytoma (DA) 3/164 (1.8%), IHG 4/164 (2.4%), ependymoma (EPN) 2/164 (1.2%), ganglioglioma (GG) 2/164 (1.2%), low grade glioma (LGG) 1/164 (0.6%), and gliosarcoma (GS) 1/164 (0.6%) (**Figure 2**, **Table S7**). Treatment details were available for 111/164 (67.7%) patients. Twenty-five out of 111 (25/111, 22.5%) patients were treated with only surgery (S) without any adjuvant treatment, these patients were defined as group 1 for subsequent multivariate analysis in the manuscript. Patients who were treated with surgery and adjuvant treatment were defined as group 2 that included 67/111 (60.4%) patients treated with surgery and adjuvant chemotherapy (S + C), 12/111 (10.8%) with surgery and targeted therapy (S+TT), 2/111 (1.8%) received surgery, chemotherapy and radiation therapy (S + C + RT) and 1/111 (0.9%) received surgery, RT and TT (S + RT + TT). Of the remaining patients, 3/111 (2.7%) received no primary treatment and 1/111 (0.9%) received chemotherapy only (**Table 1**, **Table S7**). Details of surgical extent of resection were available in 100/164 (61%) patients, 49/100 (49%) had gross total resection (GTR), 3/100 (3%) had near total resection (NTR), 31/100 (31%) had subtotal resection (STR), 15/100 (15%) had biopsy and 2/100 (2%) had no surgery (**Table 1**, **Table S7**).

**Table 1.** Clinical characteristics and treatment data

Characteristics	*n* = 164
**Gender (** *n* = **164)**	
Female	88 (53.6%)
Male	76 (46.3%)
**Tumor location (** *n* = **164)**	
Hemispheric	164 (100%)
**Age at diagnosis (months)**	
<12	120 (83.9%)
12 to <24	11 (7.7%)
24 to <36	5 (3.5%)
≥36	7 (4.9%)
NA	21
**Median follow up (months)**	33 (Range: 0-216)
NA	25
**Metastatic status**	
No	88 (94.6%)
Yes	5 (5.4%)*
NA	71
**Extent of resection**	
GTR/NTR (No Residual Disease)	52 (52%)
STR/Biopsy/No surgery (Residual Disease)	48 (48%)
NA	64
**Primary treatment (** *n* = **111)**	
Surgery + Chemotherapy	67 (60.4%)
Surgery only	25 (22.5%)
Surgery + Targeted therapy	12 (10.8%)
No treatment	3 (2.7%)
Surgery + Chemotherapy + Radiation	2 (1.8%)
Surgery + Targeted therapy +Radiation	1 (0.9%)
Chemotherapy only	1 (0.9%)
NA	53

Abbreviations: GTR: Gross Total Resection; NA: Data not available; NTR: Near Total Resection; STR: Subtotal Resection.

* Metastatic at diagnosis.

### Clinical Outcome

**Figure 4.**
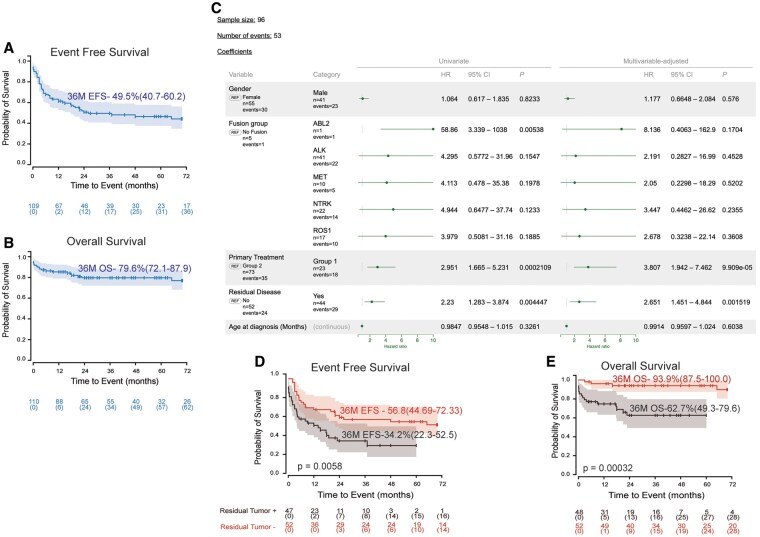
Exploring the Risk Factors in IHG. (A) Kaplan Meier EFS plot for the 109 IHG patients. (B) Kaplan Meier OS plot for 110 IHG patients. (C) Univariate and Multivariate Analysis of Prognostic Features. Group1: surgery only (S) (*n* = 23); Group 2 (*n* = 73) surgery + adjuvant therapy (Including S+C, S+TT, S+C+RT and S+TT+RT as primary treatment). HR: Hazard ratio, 95%CI : 95% confidence interval. (D) Kaplan Meier EFS plot based on presence or absence of residual disease. (E) Kaplan Meier OS plot based on presence or absence of residual disease.

Data on survival status and significant clinical events were available for 110/164 (67.1%) patients; 22/110 (20%) were reported dead at the time of the analysis. Data on follow-up were available for 110 patients with a median follow-up of 33 months (range: 0-216 months) (**Table 1**). The 3-year EFS and OS for the whole cohort was 49.5% (95% CI [40.7-60.2]) and 79.6% (95% CI [72.1-87.9]) respectively (**Figure 4A and B**). Cox regression analysis demonstrated no impact of age, gender, fusion status on EFS. Patients with residual disease were at higher risk of an event (HR: 2.651, *P* = 0.0015; **Figure 4C**). Patients who received just surgery (Group 1, *n* = 25) as treatment without adjuvant therapy were also noted to have a worse EFS (HR: 3.807) (**Figure 4C**). Similar effects were observed in OS (**Figure S3**). However, due to low event-per-variable ratio (2.4) the multivariate analysis for OS is potentially underpowered and should be interpreted with caution.

Of the 25 patients in Group 1, 7/25 (28%) did not receive adjuvant cancer directed treatment most likely due to critical clinical state and/or early death; 1/7 (14.3%) reported intra-ventricular hemorrhage, hydrocephalous and middle cerebral artery infarcts, 5/7 (71.4%) reported critical clinical condition followed by early death post-surgery and 1/7 (14.3%) reported increased intracranial pressure.

Twelve patients (12/25, 48%) in group 1 reported no progression, of them 5/12 (41.7%) were reported to have died early during the disease course due to post-operative or tumor associated complications and 1/12 (8.3%) died at 3.8 months due to severe neurological complications. Only 6/12 (50%) patients are reported alive with no progressive disease in Group 1; 5/6 (83.3%) had documented GTR post primary surgery and 1/6 (16.7%) had no information on extent of resection available.

Patients with no residual tumor were noted to have significantly better EFS and OS (*P* = 0.0058 and 0.00032, respectively) (**Figure 4D and E**). Patients who received adjuvant treatment after surgery (chemotherapy [S + C] or targeted therapy [S + TT]) had better EFS and OS (*P* = 0.026 and 0.00009, respectively) (**Figures S4E and F and S5**, **Table S8**). Sex and fusion status did not show any significant impact on EFS and OS (**Figure S4A-D**, **Table S8**). Comparison of EFS and OS in patients receiving chemotherapy (S + C) vs targeted therapy (S + TT) as primary treatment did not show significant differences (**Figures S4G-L and S5**). Overall survival of patients who received TT during their treatment course (as a primary treatment and/or post relapse treatment) was not significantly different from those that never received TT (**Figure S4O**). However, due to variability of treatment type (Entrectinib and Larotrectinib for *NTRK* and *ROS1* fusion Lorlatinib, Crizotinib and Alectinib for *ALK* fusion) and considering the era when the patients were treated (before or after clinical availability of targeted treatment), these results are more than likely to be underpowered to detect an actual effect of TT on clinical outcome and therefore, should be interpretated with caution.

#### Causes of death

**Figure 5.**
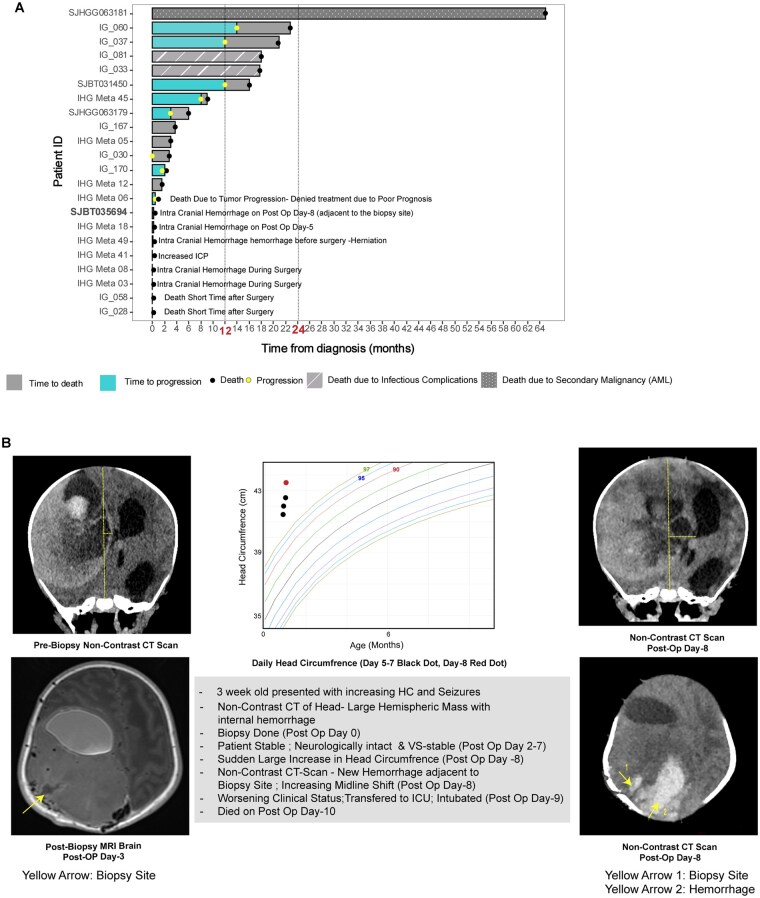
Cause and time of death in patients with IHG. (A) Swimlane plot demonstrating the 22 patients with recorded deaths within the cohort. (B) Case illustration (SJBT035694)—3-week-old with IHG died on post op day 10 due to spontaneous hemorrhage (yellow arrow—2) adjacent to the biopsy site (Yellow arrow—1). Abbreviations: ICP: intracranial pressure; HC: head circumference; ICU: intensive care unit.

There were 22 reported deaths in our cohort of 164 (22/164, 13.4%) patients with IHG. Forty-one percent (9/22, 41%) of these deaths occurred within a month of diagnosis and met the criteria for early death in oncology.^31^^,^^32^ Majority of the early deaths (5/9, 55.5%) were from intracranial hemorrhage (4/5 [80%] developed hemorrhagic complications during, or post-surgery and 1/5 [20%] died of spontaneous pre-surgical hemorrhage). Other causes of early death included progressive tumor (1/9, 11.1%), increased intracranial pressure (1/9, 11.1%) and death short time after surgery with no documented cause (2/9, 22.2%) (**Figure 5A and B** and **Table S9**).

The causes of death for all 22 patients are summarized in **Table S9**; these include progressive tumor (8/22, 36.4%), intracranial hemorrhage (5/22, 22.7%), cause for death not available (3/22, 13.6%) increased intra cranial pressure (ICP) (2/22, 9.1%), infectious complications (2/22, 9.1%), neurological deterioration (1/22, 4.5%) and secondary malignancy (1/22, 4.5%) (**Table S9**).

#### Tumor progression and salvage therapy

There were 43 patients with documented progression/relapse; median time to progression was 18 months. Post progression treatment details were available for 38 patients, 16/38 (42.1%) were treated with TT with or without surgery, 4/38 (10.5%) received surgery followed by chemotherapy, 7/38 (18.4%) and 3/38 (7.9%) got surgery or chemotherapy only, 2/38 (5.3%) got radiotherapy and 6/38 (15.8%) received no treatment post progression. There were eight second progressions reported, RT was used for salvage in 3/8 (37.5%) cases, TT was used for 4/8 (50.0%) cases and remaining two were treated with chemotherapy (**Table S10**). For four patients a third progression was reported and were treated with surgery and chemotherapy (1/4, 25.0%) and TT (3/4, 75.0%) (**Table S10**).

## Discussion

We report on a meta-analysis of 164 patients with IHG. We categorized and harmonized pertinent clinical and molecular data from 155 published and nine unpublished patients with IHG for age of diagnosis, metastatic state, extent of surgical resection, type of post-surgical adjuvant treatment, rate of progression/relapse, post progression treatment, survival status, events during the treatment and post treatment course, prevalence and type of RTK fusion, and pre-treatment primary histological diagnosis. To improve the quality and validation of published/unpublished raw data, individual patient level clinical and molecular data were obtained by directly communicating with the corresponding authors (published) and treating oncologists (unpublished) for most of the patients (*n* = 129/164) (**Table S5**). The diagnostic criteria for inclusion in the meta-analysis was adapted from the 5th edition of WHO classification of CNS tumors.^1^^,^^2^ One hundred and six tumors were confirmed to have methylation class of IHG. We found only 5% IHG tumors without any fusion in this cohort, which is much lower than the range of 20%-30% fusion negative IHGs in previously published studies. We attribute this discrepancy to higher reporting bias for RTK fusion positive tumors especially in case reports.^7–9^  *ALK* (67%) was most common gene with fusion followed by *NTRK1/2/3*, *ROS1* and *MET*. As previously reported, the IHG harbored stable genome with rare copy number variations (CNVs) and single nucleotide variants (SNVs), other than the unique RTK fusion.^7–9^

Similar to the previously published report by Chiang *et al.* on 22 patients with IHG, a large gap was noted between EFS (53.1%) and OS (90.9%).^9^ There were 43/110 documented tumor progression/relapse, 8/43 of whom were recorded as deceased, foretelling that tumor progression/relapse doesn’t necessarily determine the curability of IHG as most relapsed cases can be salvaged with subsequent therapies. A high rate of relapse despite a ∼80% OS suggests that optimum primary treatment for IHG has yet to be defined.

Predictably, patients received a broad range of post-surgical adjuvant treatment. Multiagent chemotherapy was most frequently used (60.4%), followed by TT (10.8%). A comparison of efficacy of different regimens was not feasible due to the multitudes of chemotherapy combinations and TT used. Nonetheless patients treated with adjuvant treatment (chemotherapy [S + C] or TT [S + TT]) had significantly (*P* = 0.026) better outcomes compared with those who received no adjuvant treatment post-surgery [S]. There are two possible explanations for this observation. One that patients need treatment after definitive surgery for cure in IHG; or two that patients who get critically sick post-surgery are too unstable to tolerate cancer directed treatment and succumb to hemorrhagic complications.

Presence of residual tumor was associated with higher risk of event or death than patients with no residual disease in our multivariate analysis. Other clinical and molecular factors like age at diagnosis, type of RTK fusion, or sex did not appear to affect EFS or OS. There were only five metastatic tumors in our cohort and only three patients with reported RT use during primary treatment, therefore, it was not feasible to predict the effect of metastatic disease and RT treatment on clinical outcome of newly diagnosed IHG.

Tumor relapse/progression was primarily noted early and occurred within the first two years after diagnosis. Thirty-nine patients had progression within 12-24 months and only one patient had documented late relapse (48 months), further alluding to the lack of knowledge about optimal upfront therapy for initial disease control in IHG (Table S7). A wide variety of treatment modalities were used post progression for salvage including surgery, chemotherapy and RT, although, TT with or without surgery was the most common choice of treatment after relapse.

Previously published case reports^33–35^ have alluded to the critical and potentially lethal clinical presentation of IHG during the neonatal period and in early infancy. Our study reported a 41% (9/22) early death rate (within 30 days of cancer diagnosis)^31^^,^^32^ among those patients who died (*n* = 22). This represents a much higher percentage than the 8%-9% of early deaths reported in other pediatric oncologic diagnosis. Notable causes reported in 5 of the 9 early deaths included increased intracranial pressure and spontaneous intratumoral/intra-cranial hemorrhage. Though, pre-surgical spontaneous hemorrhage was reported, most hemorrhages were related to neurosurgery. Analogously, high surgical morbidity in patients with IHG was previously reported by Chiang et al.^9^ where in a series of 30 craniotomies performed in 16 patients with IHG, 30% were complicated by high volume blood loss requiring initiation of a massive transfusion protocol and 13% had to be temporarily paused for patient stabilization.

Neurosurgical decisions at the time of presentation are often challenging not only due to the large size and hemorrhagic nature of IHG, but also because of very young age and very low body weight (< 10 kg, estimated blood volume of 70-80 ml/kg) of these patients. Furthermore, IHGs are soft friable tumors making even an attempt partial resection prone to intra-operative or postoperative bleeding that can lead to hypovolemic shock and coagulopathy. In such scenarios, the most effective means to cease bleeding is often a full resection of the tumor which is impossible to do without causing egregious harm. Therefore, unless there is a compelling need to attempt resection or debulking, a limited biopsy for accurate tissue diagnosis is often the safest option, with a preference toward open biopsy that may provide a better means to control any bleeding rather than a needle biopsy. Following, diagnostic biopsy, medical cytoreduction (chemotherapy or targeted therapy) of tumor may be considered over the course of several weeks to months- during which time the tumor volume decreases, and the infant grows, gaining the necessary weight and vitality that can support a subsequent surgery to attain GTR/NTR. Pre-surgical neoadjuvant chemotherapy has been used in limited number of cases of congenital glioblastoma with successful cytoreduction prior to primary surgery to attain GTR,^36^ yet it not used as a standard practice for IHG management. In recent years, case reports have shown that TKIs can also cause significant cytoreduction in IHG cases over the course of 2-6 months.^17^

Oncogenically driven by unique RTK fusion in an otherwise silent genome makes IHGs excellent candidates for targeted therapies with TKIs. Preclinical experiments on animal models of IHGs have shown excellent short-term efficacy and improved survival with TKI treatment,^7^^,^^8^^,^^16^ and early-phase tissue agonistic clinical trials have reported promising activity of TKIs in refractory pediatric CNS tumors with RTK fusions.^18^^,^^37^^,^^38^ Our study noted 20% TKI use as primary therapy in patients diagnosed within the last five years. In the recurrent setting, TKI was noted to be the preferred choice of treatment, with 42% of the second line treatment using targeted agents. Overall, we found a total of 26 patients who received TKI during their treatment course (primary or relapse). Most of these patients (23/26) had short follow up period (∼24 months) leaving critical questions, like long-term efficacy, optimal length of therapy with TKI, and long-term side-effects unanswered.

The authors acknowledge the selection and reporting biases associated with meta-analyses. Particularly here there was likely a reporting bias toward fusion positive IHG cases that have successfully responded to molecular directed treatments when compared to cases that did not respond well to molecular directed treatments or cases which were fusion negative and treated on standard chemotherapy. However, in the absence of disease specific clinical trials and patient registries, our manuscript is unique in providing cohesive clinically relevant information of an extremely rare tumor in statistically meaningful numbers. The authors also acknowledge that IPD pooled analysis^39^ doesn’t take into account the heterogeneity of treatment used in individual studies. Furthermore, data on molecular characteristics, staging, treatment and long-term follow up were missing in several cases and the year in which patient was diagnosed also played a role in treatment selection, since patients treated earlier did not have TKIs available as choice. For the current meta-analysis 142/164 patients were collated from case reports (*n* = 22), case series (*n* = 6), retrospective studies (*n* = 6), and clinical trial (*n* = 1) and were treated with variety of treatment regimens (**Tables S4 and S5**). The clinical trial represents 22/164 of the included patients in our study, all of them were treated on institutional protocol at St. Jude Children’s Research Hospital, Memphis, TN (**Table S4**), the EFS and OS for these 22 IHG patients have been previously reported by Chiang *et al.* in 2024 and shows a ∼37% difference between EFS and OS,^9^ which aligns with the gap in EFS and OS observed in our current analysis ∼30% (**Figure 4A and B**). We conducted a sensitivity analysis stratifying the clinical data from different sources (case series and reports, clinical trial, large retrospective cohorts) (**Figure S6A and B**), this analysis did not show significant differences in EFS and OS based on the source of the data.

Although, the results should be interpreted with extreme caution for direct clinical care, these results provide rationale and raise critical unanswered questions for future clinical trials. In summary, our meta-analysis observed: (1) high survival rates in IHGs, despite inconsistent primary treatment approaches and high rate of early progression/relapse; (2) a high incidence of early death in infants with IHG from intra-cranial hemorrhage and its associate complications; (3) an increasing trend of using TKIs both as first line and second line treatment.

Another, major drawback of the study is that it lacks data on long-term quality of life outcomes and functional outcome of the patients. Given the high-risk surgeries and use of multiple lines of treatments, it is expected that this population pays a heavy price for the high cure-rate, however the data on long-term survivorship remains limited on this population.^40^ Future, prospective trials should incorporate specific objectives studying the long-term quality of life and common morbidities in this patient population for a better understanding of the price of high cure rate.

In conclusion, our study underscores the importance of standardizing upfront treatment for this vulnerable population, providing evidence-based indication for surgery, neo-adjuvant treatment, adjuvant treatment, and use of molecularly targeted treatment in combination with or without chemotherapy. This can only be attained by well-designed stringently monitored frontline collaborative clinical trials tailored to this rare population. Such trials should focus objectives on long-term quality of life parameters along with primary treatment objectives. The current active clinical trials enrolling IHG patients are either tissue agnostic (NCT04589845) or inhibitor specific (NCT04774718 and NCT06528691) trials. Additionally, two clinical studies (NCT06333899 and NCT04655404) are testing combinations of targeted treatment with chemotherapy, but these trials are not focused specifically on IHG or infant HGG and therefore will have limited potential to standardize diagnosis specific treatment.

Currently, the only means of systematically collect IHG cases is through existing large-scale registries, or platforms, that include a variety of cancers and other genetic and non-genetic disorders contributing to childhood cancers. While these registries are helpful to understand the prevalence, treatment, and outcome of various different tumor types, the data recording systems and protocols of such platforms are generalized and often focused on the most prevalent tumor type. Therefore, these registries can miss critical details that pertain to ultra rare disease-types like IHG. By contrast a disease specific registry can capture aspects unique to a given tumor in greater depth. For example, in IHG a detailed prenatal history including prenatal ultrasound reports, perinatal history, neonatal history may be important in determining the clinical course of the patients. Therefore, an IHG specific registry that can work in collaboration with larger registries like KidsFirst and CCDI, could facilitate methodological curation of IHG pertinent data, thus providing a better clinical understanding of this tumor.

## Supplementary Material

noaf264_Supplementary_Data

## Data Availability

Data available on request from the corresponding author. All data within this manuscript are available on an interactive publicly available data portal https://viz.stjude.cloud/st-jude-childrens-research-hospital/visualization/meta-analysis-infant-type-hemispheric-gliomas∼2845.

## References

[1] Louis DN , Perry A, Wesseling P, et al. The 2021 WHO classification of tumors of the Central nervous system: a summary. Neuro Oncol. 2021;23:1231–1251. 10.1093/neuonc/noab106 34185076 PMC8328013

[2] International Agency for Research on Cancer. *WHO Classification of Tumours of the Central Nervous System: WHO Classification of Tumours*. 5th ed. WHO Classification of Tumours Editorial Board, ed. IARC; 2021.

[3] Jones C , Karajannis MA, Jones DTW, et al. Pediatric high-grade glioma: biologically and clinically in need of new thinking. Neuro Oncol. 2017;19:153–161. 10.1093/neuonc/now101 27282398 PMC5464243

[4] Sturm D , Pfister SM, Jones DTW. Pediatric gliomas: current concepts on diagnosis, biology, and clinical management. J Clin Oncol. 2017;35:2370–2377. 10.1200/JCO.2017.73.0242 28640698

[5] Mackay A , Burford A, Carvalho D, et al. Integrated molecular meta-analysis of 1,000 pediatric high-grade and diffuse intrinsic pontine glioma. Cancer Cell. 2017;32:520–537.e5. 10.1016/j.ccell.2017.08.017 28966033 PMC5637314

[6] Gielen GH , Gessi M, Buttarelli FR, et al. Genetic analysis of diffuse high-grade astrocytomas in infancy defines a novel molecular entity: molecular profiling of diffuse high-grade astrocytomas in infants. Brain Pathol. 2015;25:409–417. 10.1111/bpa.12210 25231549 PMC8029085

[7] Clarke M , Mackay A, Ismer B, et al. Infant high-grade gliomas comprise multiple subgroups characterized by novel targetable gene fusions and favorable outcomes. Cancer Discov. 2020;10:942–963. 10.1158/2159-8290.CD-19-1030 32238360 PMC8313225

[8] Guerreiro Stucklin AS , Ryall S, Fukuoka K, et al. Alterations in ALK/ROS1/NTRK/MET drive a group of infantile hemispheric gliomas. Nat Commun. 2019;10:4343. 10.1038/s41467-019-12187-5 31554817 PMC6761184

[9] Chiang J , Bagchi A, Li X, et al. High-grade glioma in infants and young children is histologically, molecularly, and clinically diverse: results from the SJYC07 trial and institutional experience. Neuro Oncol. 2024;26:178–190. 10.1093/neuonc/noad130 37503880 PMC10768990

[10] Duffner PK , Horowitz ME, Krischer JP, et al. Postoperative chemotherapy and delayed radiation in children less than three years of age with malignant brain tumors. N Engl J Med. 1993;328:1725–1731. 10.1056/NEJM199306173282401 8388548

[11] Sanders RP , Kocak M, Burger PC, Merchant TE, Gajjar A, Broniscer A. High-grade astrocytoma in very young children. Pediatr Blood Cancer. 2007;49:888–893. 10.1002/pbc.21272 17554787

[12] Geyer JR , Finlay JL, Boyett JM, et al. Survival of infants with malignant astrocytomas. A report from the childrens cancer group. Cancer. 1995;75:1045–1050. 10.1002/1097-0142(19950215)75:4<1045::aid-cncr2820750422>3.0.co; 2-k 7842407 10.1002/1097-0142(19950215)75:4<1045::aid-cncr2820750422>3.0.co;2-k

[13] Dufour C , Grill J, Lellouch-Tubiana A, et al. High-grade glioma in children under 5 years of age: a chemotherapy only approach with the BBSFOP protocol. Eur J Cancer. 2006;42:2939–2945. 10.1016/j.ejca.2006.06.021 16962317

[14] Mason WP , Grovas A, Halpern S, et al. Intensive chemotherapy and bone marrow rescue for young children with newly diagnosed malignant brain tumors. J Clin Oncol. 1998;16:210–221. 10.1200/JCO.1998.16.1.210 9440745

[15] Grundy RG , Wilne SH, Robinson KJ, et al.; Children’s Cancer and Leukaemia Group (formerly UKCCSG) Brain Tumour Committee. Primary postoperative chemotherapy without radiotherapy for treatment of brain tumours other than ependymoma in children under 3 years: results of the first UKCCSG/SIOP CNS 9204 trial. Eur J Cancer. 2010;46:120–133. 10.1016/j.ejca.2009.09.013 19818598

[16] Zuckermann M , He C, Andrews J, et al. Capmatinib is an effective treatment for MET-fusion driven pediatric high-grade glioma and synergizes with radiotherapy. Mol Cancer. 2024;23:123. 10.1186/s12943-024-02027-6 38849845 PMC11157767

[17] Bagchi A , Orr BA, Campagne O, et al. Lorlatinib in a child with ALK-Fusion–positive high-grade glioma. N Engl J Med. 2021;385:761–763. 10.1056/NEJMc2101264 34407349 PMC8672682

[18] Desai AV , Bagchi A, Armstrong AE, et al. Efficacy and safety of entrectinib in children with extracranial solid or central nervous system (CNS) tumours harbouring NTRK or ROS1 fusions. Eur J Cancer. 2025;220:115308. 10.1016/j.ejca.2025.115308 40086048

[19] Doz F , van Tilburg CM, Geoerger B, et al. Efficacy and safety of larotrectinib in TRK fusion-positive primary central nervous system tumors. Neuro Oncol. 2022;24:997–1007. 10.1093/neuonc/noab274 34850167 PMC9159442

[20] Drilon A , Laetsch TW, Kummar S, et al. Efficacy of larotrectinib in TRK fusion-positive cancers in adults and children. N Engl J Med. 2018;378:731–739. 10.1056/NEJMoa1714448 29466156 PMC5857389

[21] Veroniki AA , Hutton B, Stevens A, et al. Update to the PRISMA guidelines for network meta-analyses and scoping reviews and development of guidelines for rapid reviews: a scoping review protocol. JBI Evid Synth. 2025;23:517–526. 10.11124/JBIES-24-00308 39829235 PMC11892999

[22] Veroniki AA , Seitidis G, Tsivgoulis G, Katsanos AH, Mavridis D. An introduction to individual participant data meta-analysis. Neurology. 2023;100:1102–1110. 10.1212/WNL.0000000000207078 36797070 PMC10256124

[23] Stewart LA , Clarke M, Rovers M, et al.; PRISMA-IPD Development Group. Preferred reporting items for a systematic review and meta-analysis of individual participant data: the PRISMA-IPD statement. JAMA. 2015;313:1657–1665. 10.1001/jama.2015.3656 25919529

[24] Capper D , Jones DTW, Sill M, et al. DNA methylation-based classification of Central nervous system tumours. Nature. 2018;555:469–474. 10.1038/nature26000 29539639 PMC6093218

[25] Qaddoumi I , Orisme W, Wen J, et al. Genetic alterations in uncommon low-grade neuroepithelial tumors: BRAF, FGFR1, and MYB mutations occur at high frequency and align with morphology. Acta Neuropathol. 2016;131:833–845. 10.1007/s00401-016-1539-z 26810070 PMC4866893

[26] Chiang J , Harreld JH, Tinkle CL, et al. A single-center study of the clinicopathologic correlates of gliomas with a MYB or MYBL1 alteration. Acta Neuropathol. 2019;138:1091–1092. 10.1007/s00401-019-02081-1 31595312 PMC7467132

[27] An L , Li Y, Edmonson MN. CICERO: A versatile method for detecting complex and diverse driver fusions using cancer RNA sequencing data. *Genome Biol*. 2020;21:126. 10.1186/s13059-020-02043-x 32466770 PMC7325161

[28] Uhrig S , Ellermann J, Walther T, et al. Accurate and efficient detection of gene fusions from RNA sequencing data. Genome Res. 2021;31:448–460. 10.1101/gr.257246.119 33441414 PMC7919457

[29] Haas BJ , Dobin A, Li B, Stransky N, Pochet N, Regev A. Accuracy assessment of fusion transcript detection via read-mapping and de novo fusion transcript assembly-based methods. Genome Biol. 2019;20:213. 10.1186/s13059-019-1842-9 31639029 PMC6802306

[30] Terry M , Therneau PM. Modeling Survival Data: Extending Cox Model. Springer; 2000.

[31] Pastore G , Viscomi S, Mosso ML, et al. Early deaths from childhood cancer. A report from the childhood cancer registry of Piedmont, Italy, 1967-1998. Eur J Pediatr. 2004;163:313–319. 10.1007/s00431-004-1425-x 15346913

[32] Lind KT , Molina E, Mellies A, Schneider KW, Daley W, Green AL. Early death from childhood cancer: first medical record-level analysis reveals insights on diagnostic timing and cause of death. Cancer Med. 2023;12:20201–20211. 10.1002/cam4.6609 37787020 PMC10587965

[33] Gene-Olaciregui N , Perez-Somarriba M, Santa-María V, et al. Clinical and molecular evolution of an ALK-driven infant-type hemispheric glioma treated sequentially with second- and third-generation anaplastic lymphoma kinase inhibitors. JCO Precis Oncol. 2023;7:e2200547. 10.1200/PO.22.00547 36996378

[34] Greenwell AM , Baughan S, Altinok D, et al. Lorlatinib for the treatment of ALK Fusion-Positive Infant-Type hemispheric glioma: a case report. JCO Precis Oncol. 2022 6:e2200255. 10.1200/PO.22.00255 36315913

[35] Papusha L , Zaytseva M, Panferova A, et al. Two clinically distinct cases of infant hemispheric glioma carrying ZCCHC8: ROS1 fusion and responding to entrectinib. Neuro Oncol. 2022;24:1029–1031. 10.1093/neuonc/noac026 35196386 PMC9159448

[36] Kotecha RS , Burley K, Junckerstorff RC, et al. Chemotherapy increases amenability of surgical resection in congenital glioblastoma. Pediatr Hematol Oncol. 2012;29:538–544. 10.3109/08880018.2012.706867 22816875

[37] Desai AV , Robinson GW, Gauvain K, et al. Entrectinib in children and young adults with solid or primary CNS tumors harboring NTRK, ROS1, or ALK aberrations (STARTRK-NG). Neuro Oncol. 2022;24:1776–1789. 10.1093/neuonc/noac087 35395680 PMC9527518

[38] Bagchi A , Robinson GW, Desai AV, et al. TRLS-12. EFFICACY and safety of entrectinib in children with*ntrk* or*ros1* fusion-positive (FP) primary Central nervous system (CNS) tumors. Neuro Oncol. 2024;26:0–0. 10.1093/neuonc/noae064.165

[39] Wang H , Chen Y, Lin Y, Abesig J, Wu IX, Tam W. The methodological quality of individual participant data meta-analysis on intervention effects: systematic review. BMJ. 2021;373:n736. 10.1136/bmj.n736 33875446 PMC8054226

[40] Bagchi A , Hanzlik E, Chiang J, et al. Qol-24. NEUROLOGIC AND NEUROCOGNITIVE OUTCOMES AMONG SURVIVORS OF INFANT-TYPE HEMISPHERIC GLIOMA (IHG). Neuro Oncol. 2024;26:viii267–viii268. 10.1093/neuonc/noae165.1059

