# Supplementary material for: A systematic study of molecular diagnosis, treatment, and prognosis in infant-type hemispheric glioma: An individual patient data meta-analysis of 164 patients
Source: Neuro Oncol. 2025 Nov 8;28(3):776–89. doi: 10.1093/neuonc/noaf264 (PMC13070490; doi:10.1093/neuonc/noaf264)
Supplement: noaf264_Supplementary_Data [file noaf264_supplementary_data.zip › Supplementary_ Table_10.docx]

| **SNO.** | **Patient ID** | **Primary Treatment** | **TP-1** | **Treatment-2** | **P-2** | **TP-2** | **Treatment -3** | **P-3** | **TP-3** | **Treatment-4** | **Additional Progression** | **Status** |
| --- | --- | --- | --- | --- | --- | --- | --- | --- | --- | --- | --- | --- |
| **1** | **IHG-Meta-32** | C (HIT-MED) | NA | S + Entrectinib | No | NA | NA | NA | NA | NA | NA | Alive |
| **2** | **IG_030** | S | 0 | NA | NA | NA | NA | NA | NA | NA | NA | Dead |
| **3** | **IHG-Meta-02** | S | 0.5 | Dasatinib | No | NA | NA | NA | NA | NA | NA | Alive |
| **4** | **IHG-Meta-06** | S | 0.5 | None | No | NA | NA | NA | NA | NA | NA | Dead |
| **5** | **IG_151** | S | 1.56 | NA | NA | NA | NA | NA | NA | NA | NA | Alive |
| **6** | **IG_170** | S + C (HIT-SKK) | 2 | None (died before Rx) | NA | NA | NA | NA | NA | NA | NA | Dead |
| **7** | **SJHGG061615** | S + C (SJYC07) | 2 | S | No | NA | NA | NA | NA | NA | NA | Alive |
| **8** | **SJHGG063179** | S + C (SJYC07) | 3 | None (died before Rx) | No | NA | NA | NA | NA | NA | NA | Dead |
| **9** | **SJHGG030673** | S | 3 | None | No | NA | NA | NA | NA | NA | NA | Alive |
| **10** | **IHG-Meta-31** | S + C (POG) | 3 | S + Entrectinib | No | NA | NA | NA | NA | NA | NA | Alive |
| **11** | **mnp-20-1362** | S + C (HIT-2007) + RT | 3 | NA | No | NA | NA | NA | NA | NA | NA | Alive |
| **12** | **IHG-Meta-01** | S | 3 | Larotrectinib | No | NA | NA | NA | NA | NA | NA | Alive |
| **13** | **SJPNET030836** | S + C (SJYC07) | 4 | S + C | No | NA | NA | NA | NA | NA | NA | Alive |
| **14** | **IHG-Meta-33** | S + C (POG) | 4*** | S + Entrectinib | No | NA | NA | NA | NA | NA | NA | Alive |
| **15** | **IG_001** | S + C (Vinblastine) | 4.3 | Certinib | Yes | NA | 6TG +procarbazine + CCNU +VCR | No | NA | NA | NA | Alive |
| **16** | **IHG-Meta-47** | S + C (SJYC07) | 2.9 | S (STR) + Lorlatinib | Yes | 4.7 | Lorlatinib | No | NA | NA | NA | Alive |
| **17** | **IG_101** | S + C (CCG99703) | 4.9 | NA | NA | NA | NA | NA | NA | NA | NA | Alive |
| **18** | **IHG-Meta-17** | S | 5 | Lorlatinib | No | NA | NA | NA | NA | NA | NA | Alive |
| **19** | **IHG-Meta-22** | S + RT + Lorlatinib | 5 | S | NA | NA | NA | NA | NA | NA | NA | Alive |
| **20** | **IHG-Meta-35** | S + C (AEIOP) | 5 | Crizotinib -> Larotrectinib | No | NA | NA | NA | NA | NA | NA | Alive |
| **21** | **IHG-Meta-48** | S + C (POG) | 6 | Larotrectinib | No | NA | NA | NA | NA | NA | NA | Alive |
| **22** | **IG_102** | S | 7.2 | NA | NA | NA | NA | NA | NA | NA | NA | Alive |
| **23** | **IHG-Meta-16** | S + C (Carboplatin,Etoposide) | 8 | GTR + Lorlatinib | No | NA | NA | NA | NA | NA | NA | Alive |
| **24** | **IHG-Meta-45** | S + C | 8 | None (died before Rx) | No | NA | NA | NA | NA | NA | NA | Dead |
| **25** | **IG_156** | S + C (CG9952) | 4.1 | COG99703 | Yes | 6.1* | TMZ | Yes | 8.1* | S (STR) +methotrexate | No | Alive |
| **26** | **IHG-Meta-25** | S | 2 | TMZ + Bevacizumab + RT | Yes | 7 | Alectinib | Yes | 9 | Alectinib | 4^th^ progression at 24 months treated with Lorlatinib | Alive |
| **27** | **SJHGG031178** | S + C (SJYC07) | 8 | Entrectinib | Yes | 12 | S (GTR) + RT | No | NA | NA | NA | Alive |
| **28** | **IG_037** | S + C (POG) | 12 | TMZ+ Bevacizumab | Yes | NA | RT (Focal 59.4 Gy) | No | NA | NA | NA | Dead |
| **29** | **SJBT031450** | S + C (SJYC07) | 12 | S + Cabozantinib | No | NA | NA | NA | NA | NA | NA | Dead |
| **30** | **IG_060** | S + C (HS-III) | 14 | None (died before Rx) | No | NA | NA | NA | NA | NA | NA | Dead |
| **31** | **IHG-Meta-38** | S + C (POG) | 9 | S (STR) + C (POG) | Yes | 15 | Lorlatinib | No | NA | NA | NA | Alive |
| **32** | **SJBT031676** | S + Lorlatinib | 15 | Lorlatinib | No | NA | NA | NA | NA | NA | NA | Alive |
| **33** | **IHG-Meta-15** | S + C (CCG9921) | 19 | S + C (CCNU, TMZ) | No | NA | NA | NA | NA | NA | NA | Alive |
| **34** | **IG_057** | S + C (HS-III) | 19.9 | S | No | NA | NA | NA | NA | NA | NA | Alive |
| **35** | **SJBT034583** | S + Lorlatinib | 22 | Lorlatinib | No | NA | NA | NA | NA | NA | NA | Alive |
| **36** | **IHG-Meta-36** | S + C (AEIOP) | 22 | S + Bevacizumab + Irinotecan + RT | No | NA | NA | NA | NA | NA | NA | Alive |
| **37** | **SJHGG063177** | S + C (SJYC07) | 23 | S + C | No | NA | NA | NA | NA | NA | NA | Alive |
| **38** | **IG_087** | S + C (Myeloablative) | 26 | S (GTR) | NA | NA | NA | NA | NA | NA | NA | Alive |
| **39** | **IHG-Meta-04** | S | 24 | S (GTR) | No | NA | NA | NA | NA | NA | NA | Alive |
| **40** | **IHG-Meta-50** | S + C (POG) | 17 | S (STR) | Yes | 23 | S (STR) + RT | Yes | 28 | Larotrectinib | No | Alive |
| **41** | **IHG-Meta-13** | S + C (POG) | 2 | TMZ | Yes | 19 | Alectinib | Yes | 24 | Alectinib + Rapamycin | - 4^th^ fourth progression at 25 months, treated with surgery (NTR). - 5^th^ progression at 27 months after diagnosis treated with Lorlatinib. - 6^th^ progression at 35 months after diagnosis, treated with S (NTR) + C (TMZ) + RT. | Alive |
| **42** | **SJHGG031139** | S + Entrectinib | 37 | Lorlatinib | No | NA | NA | NA | NA | NA | NA | Alive |
| **43** | **IHG-Meta-28** | S | 48 | S (GTR) | No | NA | NA | NA | NA | NA | NA | Alive |

**Supplementary table 10** – Table of n=43 patients with progressive diseases after first line treatment. In bold are progressions. S = surgery; C = chemotherapy; TT = targeted therapy; RT = radiotherapy; NA = not assessable; GTR = gross total resection; STR = subtotal resection TMZ = temozolomide; *2 cycles of chemotherapy were counted as 2 months for time to progression (actual time data was not available); **Only a clinical progression was reported no radiological information; ***4 cycles of chemotherapy were counted as 4 months for time to progression (actual time data was not available). Time to progression is the time from diagnosis to the progression in months. TP-1 – Time to First progression in months (From Diagnosis); TP-2 – Time to Second Progression in months (From Diagnosis); TP-3- Time to Third Progression in months (From Diagnosis); P-2- 2^nd^ progression; P-3 -3^rd^ Progression*.*
