# Supplementary material for: A systematic study of molecular diagnosis, treatment, and prognosis in infant-type hemispheric glioma: An individual patient data meta-analysis of 164 patients
Source: Neuro Oncol. 2025 Nov 8;28(3):776–89. doi: 10.1093/neuonc/noaf264 (PMC13070490; doi:10.1093/neuonc/noaf264)

Supplementary Figure 3

Results

Sample size: 94

Number of events: 19

Coefficients

Variable	Category	Univariate HR	Univariate 95% CI	Univariate *P*	Multivariable-adjusted HR	Multivariable-adjusted 95% CI	Multivariable-adjusted *P*
Fusion group (REF No Fusion n=5 events=1)	ABL2 n=1 events=0	3.244e-07	0 – Inf	0.9977	7.657e-09	0 – Inf	0.9973
	ALK n=40 events=7	1.107	0.135 – 9.076	0.9246	0.2541	0.02667 – 2.42	0.2335
	MET n=10 events=3	1.948	0.2016 – 18.82	0.5645	0.375	0.03381 – 4.158	0.4243
	NTRK n=22 events=2	0.504	0.04557 – 5.573	0.5762	0.1684	0.01408 – 2.015	0.1595
	ROS1 n=16 events=6	2.26	0.2703 – 18.89	0.4517	0.5222	0.05218 – 5.225	0.5803
Primary Treatment (REF Group 2 n=71 events=10)	Group 1 n=23 events=9	4.253	1.708 – 10.59	0.00187	8.301	2.952 – 23.34	6.015e-05
Gender (REF Female n=53 events=10)	Male n=41 events=9	1.138	0.462 – 2.802	0.7791	1.417	0.5293 – 3.796	0.4877
Residual Disease (REF No n=51 events=4)	Yes n=43 events=15	6.307	2.06 – 19.32	0.001259	10.08	2.884 – 35.24	0.0002956

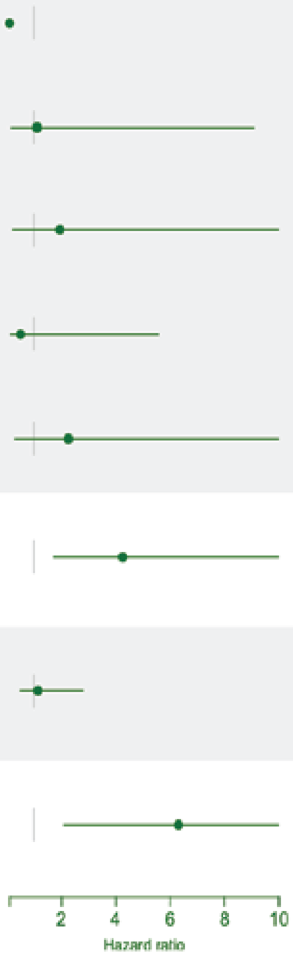

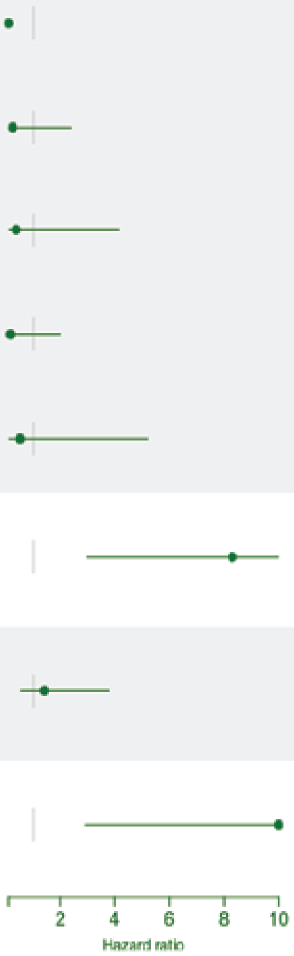

Supplement: noaf264_Supplementary_Data [file noaf264_supplementary_data.zip › Supplementary_Figure_3.pdf]
