# Supplementary material for: A systematic study of molecular diagnosis, treatment, and prognosis in infant-type hemispheric glioma: An individual patient data meta-analysis of 164 patients
Source: Neuro Oncol. 2025 Nov 8;28(3):776–89. doi: 10.1093/neuonc/noaf264 (PMC13070490; doi:10.1093/neuonc/noaf264)

Supplementary Figure 4 (continued)

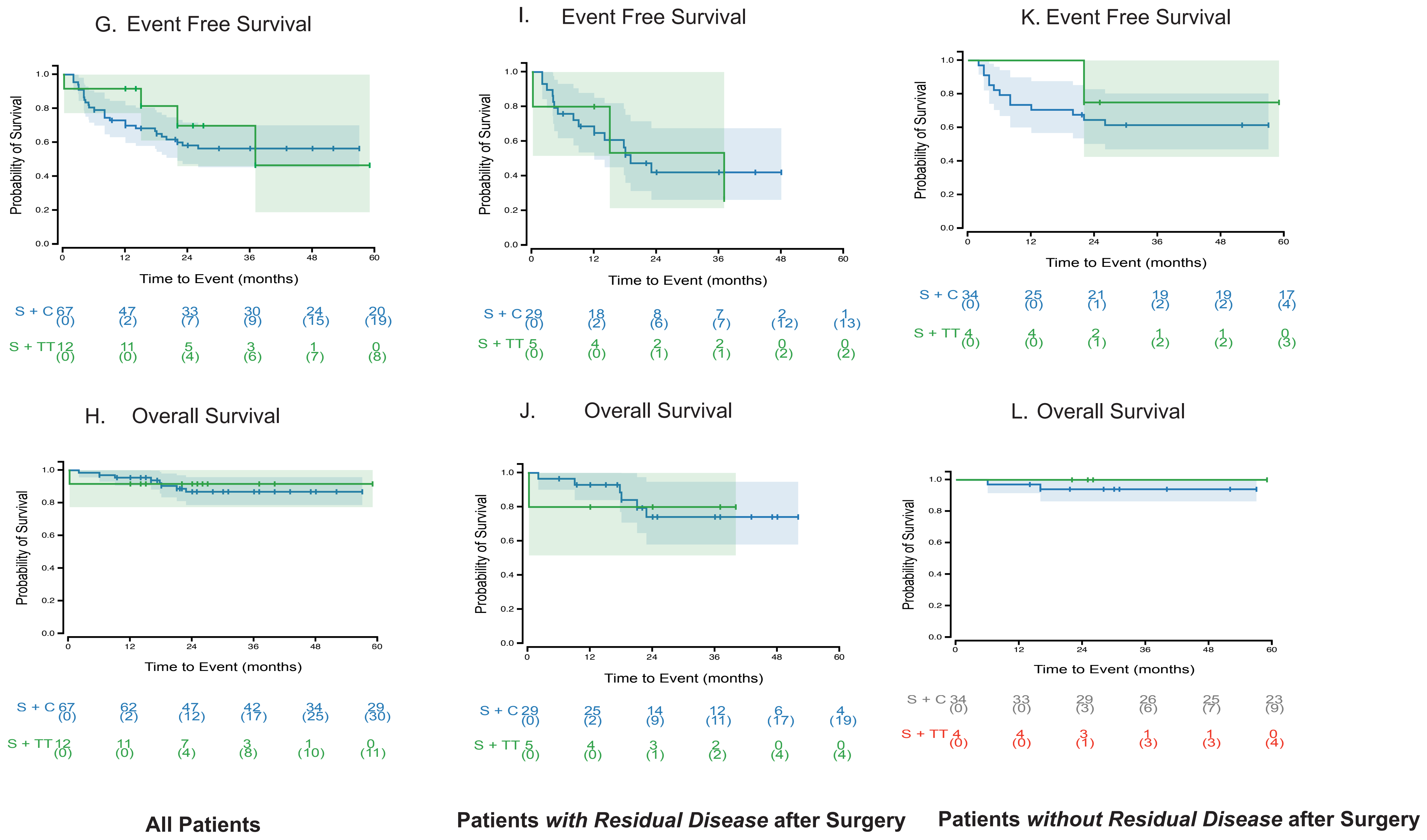

Supplement: noaf264_Supplementary_Data [file noaf264_supplementary_data.zip › Supplementary_Figure_4_(Cont).pdf]
