# Supplementary material for: A systematic study of molecular diagnosis, treatment, and prognosis in infant-type hemispheric glioma: An individual patient data meta-analysis of 164 patients
Source: Neuro Oncol. 2025 Nov 8;28(3):776–89. doi: 10.1093/neuonc/noaf264 (PMC13070490; doi:10.1093/neuonc/noaf264)
Supplement: noaf264_Supplementary_Data [file noaf264_supplementary_data.zip › Supplementary_Figure_6.pdf]

# Supplementary Figure 6

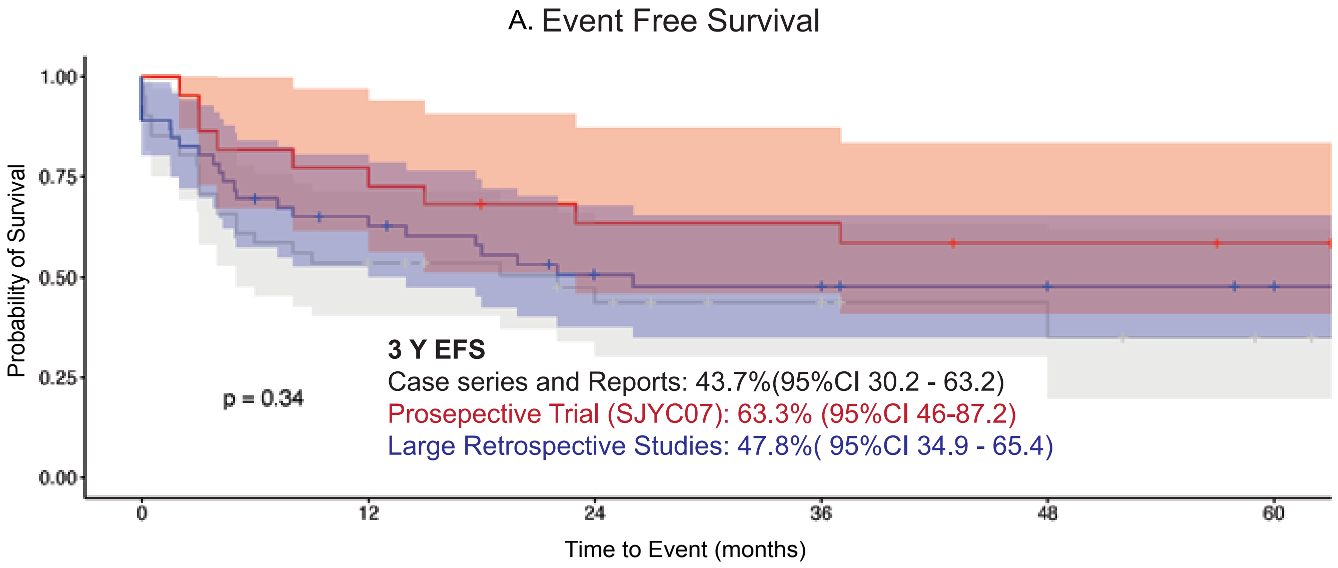


| | | | | | | |
|---|---|---|---|---|---|---|
| Case Series/Case Report | 41 | 22 | 13 | 9 | 5 | 2 |
| Prospective Trial | 22 | 17 | 13 | 13 | 11 | 10 |
| Retrospective Study | 46 | 28 | 20 | 17 | 14 | 11 |

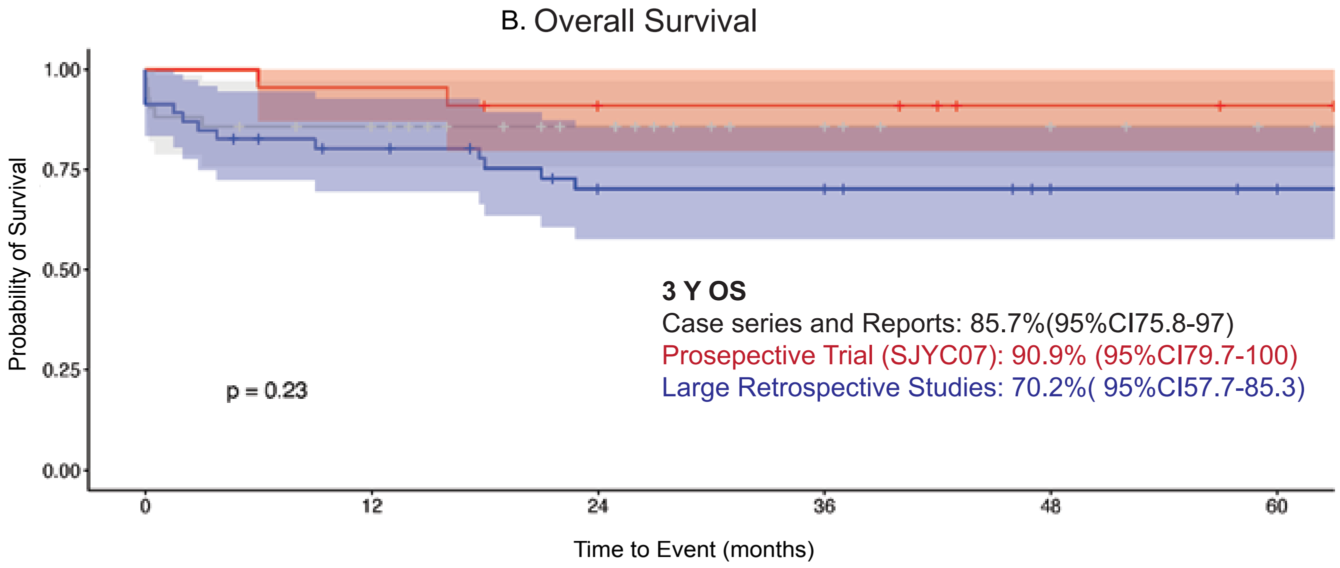


| | | | | | | |
|---|---|---|---|---|---|---|
| Case Series/Case Report | 42 | 33 | 19 | 12 | 7 | 3 |
| Prospective Trial | 22 | 21 | 19 | 18 | 14 | 13 |
| Retrospective Study | 46 | 34 | 27 | 25 | 19 | 16 |
