# Supplementary material for: A systematic study of molecular diagnosis, treatment, and prognosis in infant-type hemispheric glioma: An individual patient data meta-analysis of 164 patients
Source: Neuro Oncol. 2025 Nov 8;28(3):776–89. doi: 10.1093/neuonc/noaf264 (PMC13070490; doi:10.1093/neuonc/noaf264)
Supplement: noaf264_Supplementary_Data [file noaf264_supplementary_data.zip › Supplementary_Table1_5_8_9.docx]

**Supplementary Table 1** – Literature Search for Infant- High-Grade Glioma in Electronic Databases.

| **Electronic Database** | **Search Terms (MeSH)** | **Publications**  **(416)** |
| --- | --- | --- |
| PubMed | “Infant” AND “high-grade glioma” | 180 |
| PubMed | (Infan*) AND (hemispheric) AND (glioma*) | 127 |
| PubMed | “Infant” AND “high-grade glioma” AND “case” | 40 |
| EMBASE | Infant-type hemispheric glioma | 69 |

**Supplementary Table 2:** Studies Selected for Backward Snowballing of References (32). [Highlighted in grey, studies whose reference list has enabled new records to be identified (9).]

| **PubMed ID** | **Author** |
| --- | --- |
| 35204463 | Di Ruscio et al. |
| 31554817 | Stucklin et al. |
| 32238360 | Clarke et al. |
| 37503880 | Chiang et al. |
| 36996378 | Gene-Olaciregui et al. |
| 36315913 | Greenwell et al. |
| 38902810 | Gorodezki et al. |
| 37635016 | Sourty et al. |
| 39236755 | Simoneau et al. |
| 35196386 | Papusha et al. |
| 36707425 | Gilani et al. |
| 35404193 | Fang et al. |
| 36928815 | Strum et al. |
| 36316040 | Deland et al. |
| 37000961 | Lai et al. |
| 36601394 | Garcia et al. |
| 36910660 | Shahab et al. |
| 34407349 | Bagchi et al. |
| 33403813 | Waters et al. |
| 30220707 | Ziegler et al. |
| 35034013 | Minniti Mançano et al. |
| 37349135 | Tauziède-Espariat et al. |
| 33769175 | Kumaria et al. |
| 33900873 | Pearce et al. |
| 34910220 | Lucas et al. |
| 28966033 | Mackay et al. |
| 34626238 | Carbral de Carvalho Corrêa et al. |
| PMC916488 | Pietsche et al. |
| 38943024 | Arafah et al. |
| 38109857 | Buckner-Wolfson et al. |
| 38394779 | Tsai et al. |
| 37680912 | Yamada et al. |

**Supplementary Table 3:** Studies Identified by Backward Snowballing of References (16).

| **PubMed ID** | **Author** |
| --- | --- |
| 30709888 | Ng et al. |
| 28387643 | Kiehna et al. |
| 27121553 | Coccé et al. |
| 25795305 | Olsen et al. |
| 30350109 | Nakano et al. |
| 27530886 | Aghajan et al. |
| 30344149 | Maruggi et al. |
| 32681571 | Viaene et al. |
| 32872331 | Ceglie et al. |
| 35100688 | Hiemenz et al. |
| 32923892 | Alharbi et al. |
| 32685319 | Mrowczynski et al. |
| 31626289 | Richardson et al. |
| 33341678 | Zhong et al. |
| 31556208 | Valera et al. |
| 33738452 | Papusha et al. |

**Supplementary Table 4** – Studies Identified after database search followed by initial screening.

| **S No.** | **PubMed ID** | **Author** | **Year of Publication** | **Study Type** | **Patients Included** | **IPD**  **(Yes/No)** | **Excluded/Included** |
| --- | --- | --- | --- | --- | --- | --- | --- |
| 1 | 35204463 | Di Ruscio et al. | 2022 | Retrospective Study | 3 | Yes | Included |
| 2 | 35196386 | Papusha et al. | 2022 | Case Series | 2 | Yes | Included |
| 3 | 36707425 | Gilani et al. | 2023 | Retrospective Study | 6 | Yes | Included |
| 4 | 35404193 | Fang et al. | 2023 | Case Report | 1 | No | Included |
| 5 | 31554817 | Stucklin et al. | 2019 | Retrospective Study | 27 | Yes | Included |
| 6 | 36928815 | Strum et al. | 2023 | Retrospective Study | 5 | Yes | Included |
| 7 | 36316040 | Deland et al. | 2022 | Case Report | 1 | No | Included |
| 8 | 37000961 | Lai et al. | 2023 | Case Report | 1 | No | Included |
| 9 | 36601394 | Garcia et al. | 2022 | Case Report | 1 | No | Included |
| 10 | 36910660 | Shahab et al. | 2023 | Case Report | 1 | No | Included |
| 11 | 34407349 | Bagchi et al. | 2021 | Case Report | 0 | Yes | Excluded : Duplicate |
| 12 | 36315913 | Greenwell et al. | 2022 | Case Report | 1 | Yes | Included |
| 13 | 33403813 | Waters et al. | 2021 | Case Report | 1 | No | Included |
| 14 | 30220707 | Ziegler et al. | 2018 | Case Report | 1 | No | Included |
| 15 | 30709888 | Ng et al. | 2019 | Case Report | 1 | No | Included |
| 16 | 28387643 | Kiehna et al. | 2017 | Case Report | 1 | No | Included |
| 17 | 27121553 | Cocce et al. | 2016 | Case Report | 1 | No | Included |
| 18 | 25795305 | Olsen et al. | 2015 | Case Series | 2 | No | Included |
| 19 | 36996378 | Glen-Olaciregui et al. | 2023 | Case Report | 1 | No | Included |
| 20 | 35034013 | Mancano et al. | 2022 | Case Report | 1 | Yes | Included |
| 21 | 37349135 | Tauziede et al. | 2023 | Retrospective Study | 6 | No | Included |
| 22 | PMC9165044 | Hu et al. | 2022 | Case Series | 3 | No | Included |
| 23 | 32238360 | Clarke et al. | 2020 | Retrospective Study | 53 | Yes | Included |
| 24 | 37503880 | Chiang et al. | 2024 | Prospective Clinical Trial | 22 | Yes | Included |
| 25 | 33769175 | Kumaria et al. | 2024 | Case Report | 1 | No | Included |
| 26 | 30350109 | Nakano et al. | 2019 | Case Report | 0 | No | Exluded : Incomplete data |
| 27 | 33900873 | Pearce et al. | 2021 | Case Report | 0 | No | Exluded : Incomplete data |
| 28 | 34910220 | Lucas et al. | 2022 | Case Report | 0 | No | Exluded : Incomplete data |
| 29 | PMC9165158 | Trubicka et al. | 2022 | Case Report | 0 | No | Exluded : Incomplete data |
| 30 | 10.1093/jnen/nlac027 | Williams et al. | 2022 | Case Report | 0 | No | Exluded : Incomplete data |
| 31 | 28966033 | Mackay et al. | 2017 | Retrospective Study | 0 | Yes | Exluded : Duplicate |
| 32 | 34626238 | Carbral de Carvalho Corea et al. | 2022 | Retrospective Study | 0 | Yes | IPD : No Response |
| 33 | PMC916488 | Pietsch et al. | 2022 | Retrospective Study | 0 | Yes | IPD : No Response |
| 34 | 38943024 | Arafah et al. | 2024 | Case Report | 0 | No | Exluded : Incomplete data |
| 35 | 38109857 | Buckner et al. | 2023 | Case Series | 1 | No | Included |
| 36 | 38902810 | Gorodezki et al. | 2024 | Case Series | 2 | No | Included |
| 37 | https://doi.org/10.1093/jnen/nlae037 | Jackson et al. | 2024 | Case Report | 1 | No | Included |
| 38 | https://www.pathologyjournal.rcpa.edu.au/article/S0031-3025(23)00548-2/fulltext | Lee et al. | 2024 | Case Report | 0 | No | Exluded : Incomplete data |
| 39 | 39236755 | Simoneau et al. | 2024 | Case Report | 1 | No | Included |
| 40 | 38394779 | Tsai et al. | 2024 | Case Report | 1 | No | Included |
| 41 | 37680912 | Yamada et al. | 2023 | Case Report | 0 | No | Exluded : Incomplete data |
| 42 | 32923892 | Alharbi et al. | 2020 | Case Report | 1 | No | Included |
| 43 | 35100688 | Hiemenz et al. | 2019 | Case Report | 1 | No | Included |
| 44 | 32685319 | Mrowczynski et al. | 2020 | Case Report | 1 | No | Included |
| 45 | 33738452 | Papusha et al. | 2021 | Case Report | 1 | Yes | Included |
| 46 | 31626289 | Richardson et al. | 2019 | Case Series | 1 | No | Included |
| 47 | 31556208 | Valera et al. | 2019 | Case Report | 0 | No | Excluded : Duplicate |
| 48 | 33341678 | Zhong et al. | 2020 | Case Report | 1 | No | Included |

**Supplementary Table 5 : Individual Patient Data (IPD) ; Contact – Corresponding Authors (EFS : event free survival, OS : overall survival)**

| **PubMed ID** | **Information Requested** | **Information Not Available/ Not Shared** |
| --- | --- | --- |
| 35204463 | Molecular Data | X |
| 35196386 | Molecular Data | X |
| 36707425 | Molecular Data | X |
| 31554817 | 1. Cause of death 2. Details on primary treatment 3. Relapse/progression status 4. Details on second line treatment 5. Details on second progression 6. Molecular Data | X |
| 36928815 | 1. Age 2. Cause of death 3. Details on primary treatment 4. Relapse/progression status 5. Details on second line treatment 6. Details on second progression 7. Molecular Data | X |
| 36315913 | Molecular Data | X |
| 35034013 | 1. EFS 2. Details on primary Treatment 3. Relapse/progression status 4. Second line treatment | X |
| 32238360 | 1. Molecular Data 2. Cause of death 3. Details on primary treatment 4. Relapse/progression status 5. second line treatment | 1. Cause of death 2. Details on primary treatment 3. Relapse/progression status 4. Second line treatment |
| 37503880 | 1. EFS/OS, 2. Details on primary treatment 3. Relapse/progression status 4. second line treatment | X |
| 28966033 | Confirmation of Duplicate Cases in PMID : 32238360 | X |
| 34407349 | Confirmation of Duplicate Cases in PMID : 37503880 | X |
| 34626238 | 1. EFS/OS, 2. Details on primary treatment 3. Relapse/progression status 4. second line treatment | 1. EFS/OS, 2. Details on primary treatment 3. Relapse/progression status 4. Second line treatment |
| PMC916488 | 1. EFS/OS, 2. Details on primary treatment 3. Relapse/progression status 4. Second line treatment | 1. EFS/OS, 2. Details on primary treatment 3. Relapse/progression status 4. Second line treatment |
| 33738452 | 1.EFS  2. OS  3. Molecular Data | X |

| **Variables** | | **EFS% (95%CI)** | **p-value (Comparison)** | **OS% (95%CI)** | **p-value (Comparison)** |
| --- | --- | --- | --- | --- | --- |
| Gender | Male | 50.88 (32.29-67.61) | 0.89 (Male vs Female) | 80.19 (69.31-92.76) | 0.9 (Male vs Female) |
|  | Female | 48.48 (37.00-73.32) |  | 79.15 (69.17-90.56) |  |
| Fusion | ROS1 | 47.37 (29.49-76.09) | 0.047 (No Fusion) | 60.27 (41.07-88.44) | 0.2 (No Fusion) |
|  | MET | 42.00 (18.45-96.62) | 0.045 (No Fusion) | 66.67 (41.53-100.0) | 0.32 (No Fusion |
|  | NTRK | 36.89 (21.14-64.37) | 0.028 (No Fusion) | 91.30 (80.49-100.0) | 0.83 (No Fusion) |
|  | ALK | 51.22 (38.39-68.33) | 0.069 (No Fusion) | 82.55 (72.26-94.31) | 0.64 (No Fusion) |
|  | No Fusion | 100.00 (100.0-100) | ------- | 100.00 | ------- |
| Primary Treatment | S + C | 56.43 (45.41-70.13) | 0.00009 (S) | 86.85 (78.72-95.83) | 0.0008 (S) |
|  | S | 28.57 (15.04-54.27) | ---- | 62.50 (45.85-85.21) | ----- |
|  | S + TT | 69.84 (46.02-100) | 0.026 (S) | 91.67 (77.29-100) | 0.08 (S) |
| Primary Adjuvant Treatment **(All Patients)** | S +C | 56.43 (45.41-70.13) | 0.62 | 86.85 (78.72-95.83) | 0.83 |
|  | S+TT | 69.84 (46.02-100) |  | 91.67 (77.29-100) |  |
| Primary Adjuvant Treatment (**Residual Disease)** | S+C | 42.1 (26.2-67.5) | 0.92 | 74.1 (58.0-94.7) | 0.97 |
|  | S+TT | 53.3 (21.4-100.0) |  | 80.0 (51.6-100) |  |
| Primary Adjuvant Treatment **(No Residual Disease)** | S+C | 61.5 (47.1-80.3) | 0.57 | 94.0 (83.3-100.00) | 0.62 |
|  | S+TT | 75.0 (42.6-100.00) |  | 100.0 |  |
| Patients who received TT vs no TT | No TT |  |  | 75.6 (66.6-85.8) | 0.071 |
|  | TT |  |  | 91.6 (80.8-100.0) |  |
| Patients who received S+C as primary treatment. | CCE | 71.4 (44.7-100) |  | 85.7 (63.3-100) |  |
|  | HDC +SCR | 20.8 (3.7-100) |  | 60 (29.3-100) |  |
|  | HIT SKK | 80 (51-100) |  | 80 (51.6-100) |  |
|  | Miscellaneous | 56.2 (36.5-86.7) |  | 100 (50.3-100) |  |
|  | POG like | 31.6 (13-76.9) |  | 75 (0-100) |  |
|  | SJYC07 Like | 69.6 (52-93.2) |  | 94.7 (85.2-100) |  |

**Supplementary Table 8: 36 Months EFS and OS;** EFS: event-free survival; OS: overall survival; 95%CI: 95% confidence interval; S: Surgery; S + C: Surgery + Chemotherapy; S + TT: Surgery +Targeted therapy; TT: Targeted therapy; CCE: Carboplatin + Etoposide +/- Cyclophosphamide; HDC +SCR: High Dose Chemotherapy with Stem Cell Rescue; POG like: Cisplatin +Vincristine + Cyclophosphamide; SJYC07 Like: High Dose Methotrexate, Cisplatin, Vincristine, Cyclophosphamide.

| Patient ID | PMID | Age at diagnosis (months) | OS (months) | Primary treatment | Cause of death |
| --- | --- | --- | --- | --- | --- |
| IG_028 | 31554817 | 1.4 | 0 | Surgery (biopsy) | Patient died within short time after primary surgery.  IPD form indicated progressive disease, given EFS=OS= 0 months we do not consider it as a progression |
| IG_058 | 31554817 | 0.0 | 0 | Surgery (biopsy) | Patient died within short time after primary surgery.  IPD form indicated progressive disease, given EFS=OS= 0 months we do not consider it as a progression |
| IHG-Meta-03 | 27121553 | 2.0 | 0 | None | Intra cranial hemorrhage during primary surgery |
| IHG-Meta-08 | 36707425 | 3.0 | 0 | None | Intra cranial hemorrhage during primary surgery |
| IHG-Meta-41 | 37349135 | 2.0 | 0 | Surgery (biopsy) | Increased intra cranial pressure |
| IHG-Meta-49 | 33341678 | 0.0 | 0.1 | NA | Died before Surgery due herniation from Tumor volume and Intra-cranial Hemorrhage (Congenital Tumor) |
| IHG-Meta-18 | 35100688 | 1.6 | 0.2 | Surgery (STR) | Intra cranial hemorrhage on post-operative day 5 |
| SJBT035694 | New Case-6 | 1 | 0.2 | Surgery (biopsy) + Lorlatinib | Intra cranial hemorrhage adjacent to the biopsy site on Day-8 during Lorlatinib treatment. |
| IHG-Meta-06 | 36601394 | 0 | 0.5 | Surgery (biopsy) | Tumor Progression (Denied therapy due to poor prognosis) |
| IHG-Meta-12 | 36707425 | 0 | 1.5 | Surgery (biopsy) | Patient just had Biopsy and EVD placed, died before any treatment could be started. No reason of death documented but could be of progressive disease |
| IG_170 | 31554817 | 0 | 2 | Surgery (biopsy) + Chemotherapy (HIT SKK) | Tumor Progression |
| IG_030 | 31554817 | 1.6 | 2.3 | Surgery (STR) | Tumor Progression |
| IHG-Meta-05 | 35404193 | 4 | 3.0 | Surgery (GTR) | 3 Months after Surgery during Chemotherapy (Reason not known) |
| IG_167 | 31554817 | 3 | 3.8 | Surgery (STR) | Neurologic deterioration due to tumor complication (herniation at diagnosis) |
| SJHGG063179 | 37503880 | 1.30 | 6 | Surgery (GTR) + Chemotherapy (MTX, cyclophosphamide, cisplatin, vincristine) | Tumor Progression |
| IHG-Meta-45 | 37349135 | 6 | 9.0 | Surgery (STR) + Chemotherapy (no details available) | Tumor Progression |
| SJBT031450 | 37503880 | 6.60 | 16 | Surgery (GTR) + Chemotherapy (MTX, cyclophosphamide, cisplatin, vincristine) | Tumor Progression |
| IG_033 | 31554817 | 3.9 | 17.7 | Surgery (STR) + Chemotherapy (POG) | Infection-Sepsis (patient was admitted to ICU for fever and neutropenia, and the likely cause of death was noted to be sepsis). |
| IG_081 | 31554817 | 1.40 | 18 | Surgery (STR) + Chemotherapy (Myeloablative with SCR) | Infection, streptococcal Meningitis (vasculitis, brain stem and BG infarction, streptococcal meningitis and Klebsiella sepsis) |
| IG_037 | 31554817 | 4.70 | 21 | Surgery (STR) + Chemotherapy (POG) | Tumor Progression |
| IG_060 | 31554817 | 10 | 22.8 | Surgery (biopsy) + Chemotherapy (Headstart3) | Tumor Progression |
| SJHGG063181 | 37503880 | 3,.0 | 65 | Surgery (GTR) + Chemotherapy (MTX, cyclophosphamide, cisplatin, vincristine) | Secondary AML |

**Supplementary Table 9: Causes of death in 22 patients.**

Highlighted in grey are the cases with early deaths (< month from diagnosis)

IPD: individual patient data; EFS: event-free survival; OS: overall survival; STR: Subtotal Resection; GTR: Gross Total Resection; NTR: Near Total Resection; MTX: Methotrexate; SCR: Stem Cell Rescue; AML: Acute Myeloid Leukemia.
